# Systematic Review of the Empirical Evidence of Study Publication Bias and Outcome Reporting Bias — An Updated Review

**DOI:** 10.1371/journal.pone.0066844

**Published:** 2013-07-05

**Authors:** Kerry Dwan, Carrol Gamble, Paula R. Williamson, Jamie J. Kirkham, the Reporting Bias Group

**Affiliations:** Department of Biostatistics, University of Liverpool, Liverpool, England; University Paris Descartes, France

## Abstract

**Background:**

The increased use of meta-analysis in systematic reviews of healthcare interventions has highlighted several types of bias that can arise during the completion of a randomised controlled trial. Study publication bias and outcome reporting bias have been recognised as a potential threat to the validity of meta-analysis and can make the readily available evidence unreliable for decision making.

**Methodology/Principal Findings:**

In this update, we review and summarise the evidence from cohort studies that have assessed study publication bias or outcome reporting bias in randomised controlled trials. Twenty studies were eligible of which four were newly identified in this update. Only two followed the cohort all the way through from protocol approval to information regarding publication of outcomes. Fifteen of the studies investigated study publication bias and five investigated outcome reporting bias. Three studies have found that statistically significant outcomes had a higher odds of being fully reported compared to non-significant outcomes (range of odds ratios: 2.2 to 4.7). In comparing trial publications to protocols, we found that 40–62% of studies had at least one primary outcome that was changed, introduced, or omitted. We decided not to undertake meta-analysis due to the differences between studies.

**Conclusions:**

This update does not change the conclusions of the review in which 16 studies were included. Direct empirical evidence for the existence of study publication bias and outcome reporting bias is shown. There is strong evidence of an association between significant results and publication; studies that report positive or significant results are more likely to be published and outcomes that are statistically significant have higher odds of being fully reported. Publications have been found to be inconsistent with their protocols. Researchers need to be aware of the problems of both types of bias and efforts should be concentrated on improving the reporting of trials.

## Introduction

Study publication bias arises when studies are published or not depending on their results; it has received much attention [1], [2]. Empirical research consistently suggests that published work is more likely to be positive or statistically significant (P<0.05) than unpublished research [3]. Study publication bias will lead to overestimation of treatment effects; it has been recognised as a threat to the validity of meta-analysis and can make the readily available evidence unreliable for decision making. There is additional evidence that research without statistically significant results takes longer to achieve publication than research with significant results, further biasing evidence over time [4]–[7]. This “time lag bias” (or “pipeline bias”) will tend to add to the bias since results from early available evidence tend to be inflated and exaggerated [8], [9].

Within-study selective reporting bias relates to studies that have been published. It has been defined as the selection on the basis of the results of a subset of the original variables recorded for inclusion in a publication [10]. Several different types of selective reporting within a study may occur. For example, selective reporting of analyses may include intention-to–treat analyses versus per–protocol analyses, endpoint score versus change from baseline, different time points or subgroups [11]. Here we focus on the selective reporting of outcomes from those that were originally measured within a study; outcome reporting bias (ORB).

Randomised controlled trials (RCTs) are planned experiments, involving the random assignment of participants to interventions, and are seen as the gold standard of study designs to evaluate the effectiveness of a treatment in medical research in humans [12]. The likely bias from selective outcome reporting is to overestimate the effect of the experimental treatment.

The original version of this systematic review [13] summarised the empirical evidence for the existence of study publication bias and outcome reporting bias. It found that 12 of the 16 included empirical studies demonstrated consistent evidence of an association between positive or statistically significant results and publication and that statistically significant outcomes have higher odds of being fully reported.

The ORBIT (Outcome Reporting Bias In Trials) study conducted by authors of this review, found that a third of Cochrane reviews found at least one trial with high suspicion of outcome reporting bias for a single review primary outcome [14]. Work has also been published to show how to identify outcome reporting bias within a review and relevant trial reports [15].

Studies comparing trial publications to protocols or trial registries are also accumulating evidence on the proportion of studies in which at least one primary outcome was changed, introduced, or omitted [16].

Thus, the bias from missing outcome data that may affect a meta-analysis is on two levels: non-publication due to lack of submission or rejection of study reports (a study level problem) and the selective non-reporting of outcomes within published studies on the basis of the results (an outcome level problem). While much effort has been invested in trying to identify the former [1], [2], it is equally important to understand the nature and frequency of missing data from the latter level.

The aim of this study was to update the original review [13] and summarise the evidence from empirical cohort studies that have assessed study publication bias and/or outcome reporting bias in RCTs approved by a specific ethics committee or other inception cohorts of RCTs.

## Methods

### Study Inclusion Criteria

We included research that assessed an inception cohort of RCTs for study publication bias and/or outcome reporting bias. We focussed on inception cohorts with study protocols being registered before the start of the study as this type of prospective design were deemed more reliable. We excluded cohorts based on prevalence archives, in which a protocol is registered after a study is launched or completed, since such cohorts can already be affected by publication and selection bias.

Both cohorts containing exclusively RCTs or containing a mix of RCTs and non-RCTs were eligible. For those studies where it was not possible to identify the study type (i.e. whether any included studies were RCTs), we attempted to contact the authors to try to resolve this. In cases where it could not be resolved, studies were excluded. Those studies containing exclusively non-RCTs were excluded.

The assessment of RCTs in the included studies had to involve comparison of the protocol against all publications (for outcome reporting bias) or information from trialists (for study publication bias).

### Search Strategy

The search strategy from the original version of this review [13] was used in this update. In the original review, screening of titles was carried out by one author (KD), but in this update two authors (KD and JJK) screened both titles and abstracts. No masking was used during the screening of abstracts. MEDLINE (1946 to 2012), SCOPUS (1960 to 2012) and the Cochrane Methodology Register (1898 to 2012) were searched without language restrictions (see Appendix S1 for all search strategies). SCOPUS is a much larger database than EMBASE, it offers more coverage of scientific, technical, medical and social science literature than any other database. Over 90% of the sources indexed by EMBASE are also indexed by SCOPUS plus many other indexed sources as well.

Additional steps were taken to complement electronic database searches:the lead or contact authors of all identified studies were asked to identify further studies and references of included studies were checked for further eligible studies.

### Quality Assessment

To assess the methodological quality of the included studies, the same criteria was applied as in the original version of this review [13].

1. Was there an inception cohort?

Yes = a sample of clinical trials registered at onset or on a roster (e.g. approved by an ethics committee) during a specified period of time.

No = anything else.

Unclear.

2. Was there complete follow up (after data-analysis) of all the trials in the cohort?

Yes ≥90%.

No <90%.

Unclear.

3. Was publication ascertained through personal contact with the investigators?

Yes = personal contact with investigators, or searching the literature and personal contact with the investigator.

No = searching the literature only.

Unclear.

4. Were positive and negative findings clearly defined?

Yes = clearly defined.

No = not clearly defined.

Unclear.

5. Were protocols compared to publications?

Yes = protocols were compared to publications.

No = protocols were not considered in the study.

Unclear.

### Data Extraction

**Figure 1:**
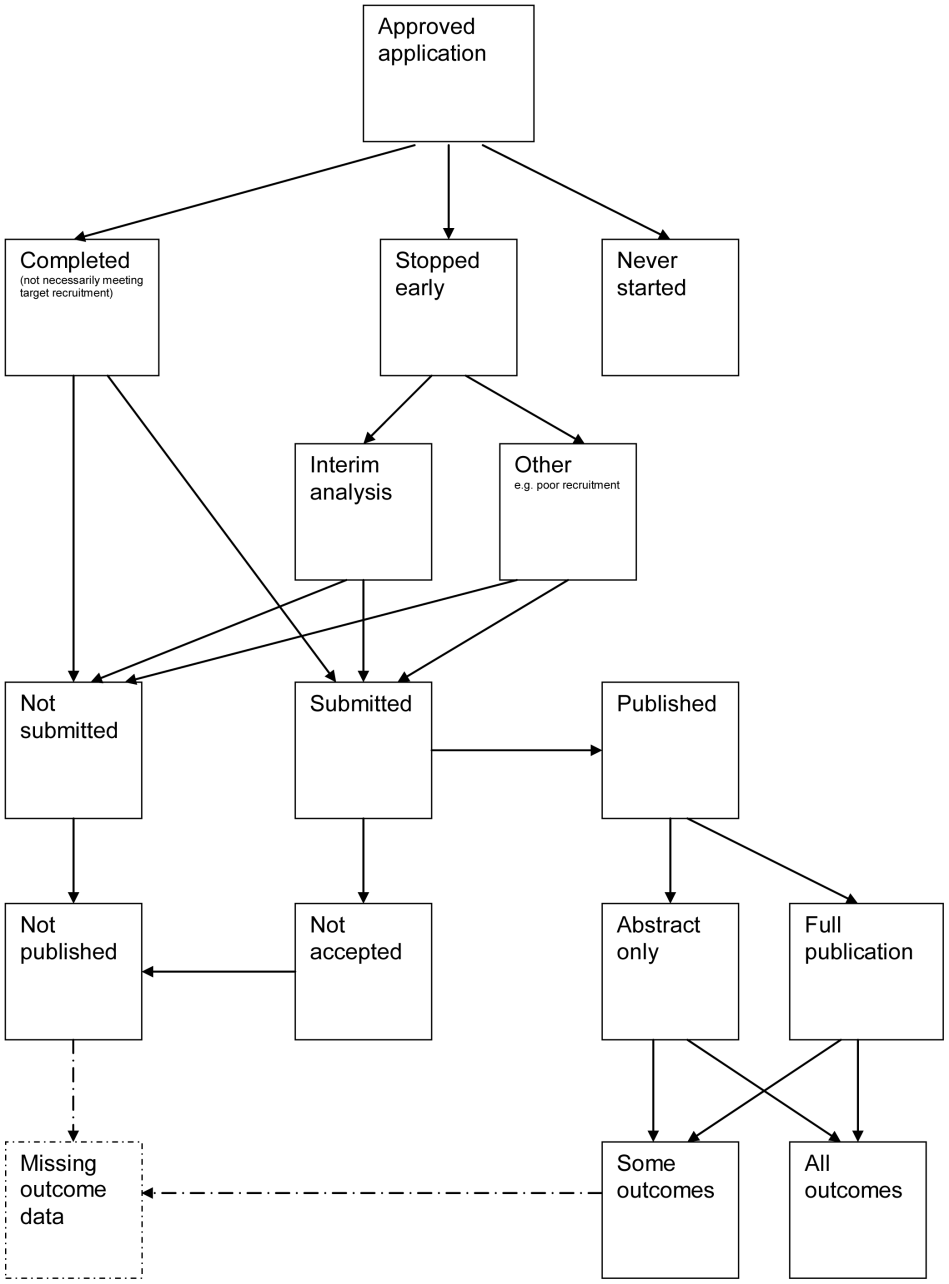
Study flow diagram.

A flow diagram (Figure 1, text S1) to show the status of approved protocols was completed for each empirical study by the first author only (KD) in the original version of the review and by two authors in the update (KD and JJK) using information available in the publication or further publications. Disagreements were resolved through discussion. Lead or contact authors of the empirical studies were then contacted by email and sent the flow diagram for their study to check the extracted data along with requests for further information or clarification of definitions if required. No masking was used and disagreements were resolved through discussion between KD and the lead or contact author of the empirical studies. Where comments from the original author were not available, PRW reviewed the report and discussed queries with KD in the original version of the review.

Characteristics of the cohorts were extracted by the first author in the original version of the review for each empirical study and issues relating to the methodological quality of the study were noted. This process was undertaken by two authors (JJK and KD) for newly identified studies in the update of this review. We recorded the definitions of ‘published’ employed in each empirical study. Further, we looked at the way the significance of the results of the studies in each cohort were investigated (i.e. direction of results and whether the study considered a *p*-value ≤0.05 as definition of significance and where there were no statistical tests whether the results were categorised as negative, positive, important or unimportant). We extracted data on the number of positive and negative trials that were published in each cohort and we extracted all information on the main objectives of each empirical study and separated these according to whether they related to study level or outcome level bias.

### Data Analysis

This review provides a descriptive summary of the included empirical studies. We refrained from statistically combining results from the different cohorts due to the differences in their design.

## Results

### Search Results

The search of MEDLINE, SCOPUS and the Cochrane Methodology Register led to 2525, 2090 and 832 references, respectively. Titles were checked by the two authors (KD and JJK) in this update and abstracts obtained for 86 potentially relevant studies. Abstracts were assessed for eligibility by both authors; 40 were excluded and full papers were obtained when available for 46.

Nineteen empirical studies were deemed eligible [3]–[5], [7], [17]–[31], sixteen of which were included in the original version of this review [13].

References from the included empirical studies led to another eligible study [32].

**Figure 2:**
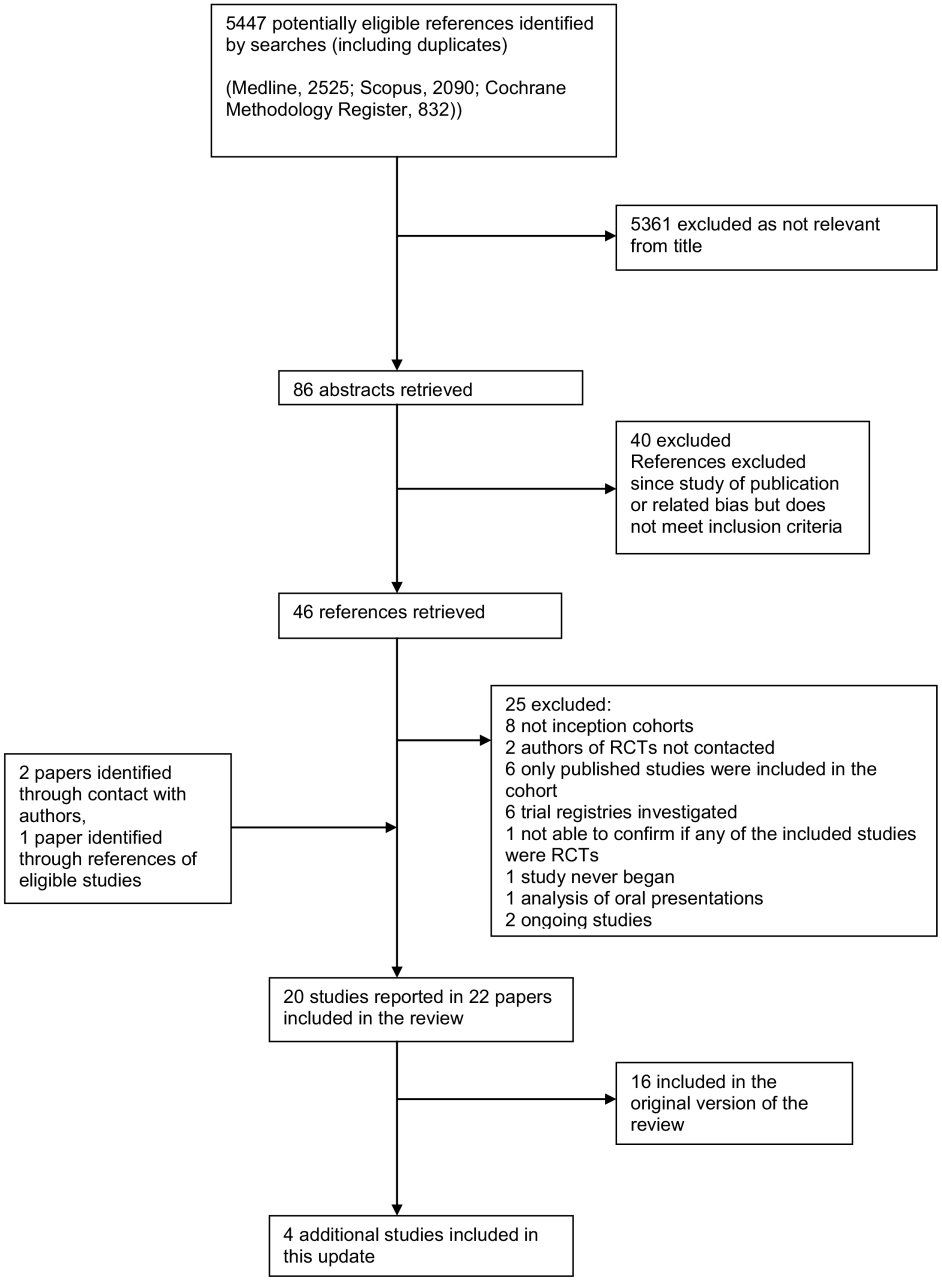
PRISMA flow diagram.

Thus in total, the search strategy identified 20 eligible empirical studies (Figure 2), of which, four were newly included in this update [21], [28], [31], [32]. Two studies that should have been in the original review were included in this review. One study [28] was missed as a result of single author study selection in the original review and a second study [32] was identified through a reference search of a newly identified study [21]. All previously identified studies were found again.

Two further studies are ongoing [33], [34].

### Excluded Studies

Twenty five studies were excluded; eight were not inception cohorts [35]–[42]; in two studies, the authors of included RCTs were not contacted for information on publication [43], [44]; in six, only published studies were included in the cohort [45]–[50]; in six studies, trial registries were investigated and were not considered as inception cohorts [51]–[56]; in one we could not confirm if any of the included studies were RCTs [57]; the author of a letter confirmed the study mentioned never began [58] and in a further study [59] was an analysis on oral presentations from one of the included studies [7].

### Included Studies

#### Study publication bias

Fifteen empirical studies considered the process up to the point of publication [3]–[5], [7], . However, six of these empirical studies [20], [21], [27], [28], [31], [32] did not consider whether a study was submitted for publication.

Five cohorts included only RCTs [3], [5], [27], [28], [30]; in the remaining ten cohorts [4], [7], [19]–[24], [31], [32] the proportion of included RCTs ranged from 14% to 56%. The results presented in the flow diagrams relate to all studies within each cohort because it was not possible to separate information for different types of studies (RCTs versus other).

#### Outcome reporting bias

Five empirical studies covered the entire process from the study protocol to the publication of study outcomes [17], [18], [25], [26], [29]. However, three of these empirical studies [25], [26], [29] did not consider whether a study was submitted for publication. Four cohorts included only RCTs [17], [18], [25], [29]; in the remaining cohort [26] the proportion of included RCTs was 13%.

Two studies are currently being updated and data on outcomes is being analysed for publication [29], [31].

### Study Characteristics

Table 1 contains information on empirical study characteristics. The majority of the empirical study objectives related to study publication bias and publication rates or outcome reporting bias.

**Table 1:** Study characteristics for inception cohorts.

Study	Objective	Committee approving protocols (country)	Period protocols approved	Date of follow up	Included study designs; Number of studies/Total number of studies (percentage of studies included)	Funding source for all studies	Conclusions
Easterbrook, 1991 [24]	**Study publication bias:** Evidence of publication bias	Central Oxford research Ethics committee (UK)	1984–1987	1990	Analysed^1^: RCTs 148/285 (52%), observational 86/285 (30%), non-RCT 51/285 (18%)	17% unfunded, 20% NHS or department, 13% Government, 38% Pharmaceutical industry, 12% private/charity	Studies with statistically significant results were more likely to be published, also more likely to lead to a greater number of publications and presentations and to be published in journals with a high citation impact factor.
Dickersin, 1992 [23]	**Study publication bias:** To investigate factors associated with the publication of research findings, in particular, the association between ‘significant’ results and publication.	Institutional Review Boards that serve The John Hopkins Health Institutions (USA)	1980	1988	Completed^2^: RCTs 168/514 (33%), observational 273/514 (53%), other experimental 73/514 (14%)	45% NIH, 12% other government, 8% Drug industry, 63% Other, 4% Internal, 18% None.	There is a statistically significant association between significant results and publication.
Dickersin, 1993 [3]	**Study publication bias:** To investigate the association between trial characteristics, findings and publication.	National Institutes of Health (USA)	1979	1988	RCTs 310/310 (100%)	50% Grant, 30% Contract, 20% Intramural.	Publication bias is a significant problem
Stern, 1997 [4]	**Study publication bias and time lag bias:** To determine the extent of publication bias and whether publication was delayed for studies with negative results in comparison with those with positive results.	Approved Royal Prince Alfred hospital ethics committee application (Australia)	1979–1988	1992	Total: RCTs 418/748 (56%), observational 165/748 (22%), non trial experiment 165/748 (22%) Completed questionnaires: RCTs 277/520 (53%), observational 129/520 (25%), non trial experiment 114/520 (22%) Analysed^3^: RCTs 167/321 (52%), observational 90/321 (28%), non trial experiment 64/321 (20%)	117/321 Internal, 206/321 External	Confirms the evidence of publication bias found in other studies. Identifies delay in publication as an additional important factor
Cooper, 1997 [19]	**Study publication bias:** To determine the fate of studies approved by their departmental human subjects review committee	Department of Psychology Human Subjects Committee or Institutional Review Board, Midwestern, research oriented, state university, USA	1986–1988	NI	NI	NI	Significant findings were more likely than non-significant findings to be submitted for meeting presentation or publication.
Wormald, 1997 [30]	**Study publication bias:** To determine the outcome of all randomised controlled trials processed through the pharmacy of Moorfields eye hospital and to determine whether the publication status of these trials is associated with observed effect of treatment.	Trials processed through the pharmacy of Moorfields Eye Hospital (UK)	1963–1995	1997	RCTs 61/61 (100%)	NI	There was limited evidence of publication bias
Ioannidis, 1998 [5]	**Study publication bias and time lag bias:** To evaluate whether the time to completion and time to publication of randomized phase 2 and phase 3 trials are affected by the statistical significance of results.	Efficacy clinical trials conducted by AIDS Clinical Trials Group and Terry Beirn Community Programs for Clinical Research on AIDS (USA)	1986–1996	1996	RCTs 109/109 (100%)	Data managed by: 10% Pharmaceutical industry, 90% Other federally sponsored.	There is a time lag in the publication of negative findings that occurs mostly after the completion of the trial follow up.
Pich, 2003 [27]	**Publication rate:** To assess the outcome of protocols submitted to the HCEC.	Hospital Clinic Ethics Committee (Spain)	1997	2001	RCTs 158/158 (100%)	89% Pharmaceutical industry, 11% Other.	Only 64% of trials that started were finally implemented and finished in accordance with the original protocol. Only 31% of closed clinical trials were published or in-press in peer reviewed journals.
Cronin, 2004 [20]	**Study publication bias:** Assess the degree to which research project findings were published and explore factors that influenced publication	R&D projects funded by the NHS and commissioned by the North Thames Regional Office (UK)	1993–1998	1995–1998	NI	100% government	Funders should consider the significant number of studies that did not result in publication and the higher rate of publication in peer reviewed journals from some programs
Decullier, 2005 [7]	**Study publication bias and time lag bias:** To describe the fate of approved protocols and assess publication bias at a national level.	French Research Ethics Committees (France)	1994	2000–2002	Total: RCTs 345/649 (53%), descriptive/observational 91/649 (14%), non-randomised 213/649 (33%) Completed: RCTs 269/501 (54%), descriptive/observational 66/501 (13%), non-randomised 166/501 (33%)	8% No funding, 73% Private funding, 13% Public, 6% Mixed.	Too many studies are not completed and too many are not published.
Decullier, 2006 [22]	**Study publication bias:** To investigate the fate of protocols submitted for funding, whether they were funded or not.	Greater Lyon regional scientific committee (France)	1997	2003	RCTs 20/142 (14%), experimental 15/142 (10%), descriptive 45/142 (32%), analytical 27/142 (19%), not clinical 28/142 (20%), not available 7/142 (5%)	38% committee funded	Some protocols submitted for funding were initiated and completed without any funding declared. To our understanding this means that not all protocols submitted really needed funding and also that health care facilities are unaware that they implicitly financially support and pay for biomedical research.
Hahn, 2002 [26]	**Outcome reporting bias:** To examine the extent of within-study selective reporting in clinical research	Local Research Ethics Committee (UK)	1994	1999	Of 15 published: RCTs 2/15 (13%), non RCT 2 (13%), uncontrolled trial 2 (13%), case control 1 (7%), survey 2 (13%), cohort and case control 1 (7%), method evaluation study 5 (34%)	Not recorded	Within-study selective reporting may be examined qualitatively by comparing the study report with the protocol. The results suggest that it might well be substantial; the bias could only be broadly identified as protocols were not sufficiently precise.
Chan, 2004a [18]	**Outcome reporting bias:** To determine whether outcome reporting bias would be present in a cohort of government funded trials subjected to rigorous peer review.	Canadian Institutes of Health Research (Canada)	1990–1998	2002/2003	RCTs 108/108 (100%)	42% jointly funded by industry and CIHR/MRC, 58% no industry funding.	Selective reporting of outcomes frequently occurs in publications of high-quality government-funded trials.
Chan, 2004b [17]	**Outcome reporting bias:** To study empirically the extent and nature of outcome reporting bias in a cohort of RCTs	Scientific-Ethical Committees for Copenhagen and Frederiksberge, Denmark	1994–1995	2003	RCTs 304/304 (100%)	55% Full industry, 17% Partial industry, 22% Non-industry, 7% non declared.	Reporting of trials is frequently incomplete, biased and inconsistent with protocols.
Ghersi, 2006 [25]	**Outcome reporting bias:** To identify discrepancies in the identity and definition of the primary outcome and to investigate factors associated with the completeness of reporting of the primary outcome.	CSAHS Ethics review committee (Australia)	1992–1996	NI	RCTs 318/318 (100%)	37% commercial funding, 63% no commercial funding.	NI
Von Elm, 2008 [29]	**Outcome reporting bias:** To study trial outcomes specified in protocols of RCTs and reported in subsequent full publications and to estimate publication rate. Investigate whether outcomes are discrepant and to investigate factors that are associated with complete reporting (e.g. statistical significance, funding)	University of Berne/CH ethics committee (Switzerland)	1988–1998	2006	Total: RCTs 451/1698 (27%) In depth analyses: 451/451 (100%)	81% industry, 10% other^4^,	About half of drug trials are not published. A high prevalence of pre-specified outcomes are not reported and discrepancies includes primary outcomes. Completeness of reporting of an outcome is associated with statistical significance.
Turer 2007 [28]	**Publication rate: To assess the non publication of clinical research**	Duke University Health System IRB (USA)	1998	2005	RCTs 369/369 (100%)	16% government, 77% industry, 3% internal, 4% independent trial group	Results of almost half of the multicenter clinical trials conducted in part at a large academic medical centre have never been published. Mechanisms to ensure public dissemination of clinical trial results are needed.
De jong 2010 [21]	**Publication rate: To determine the percentage of unpublished studies approved by the REC of a major Dutch academic medical center and to identify factors associated with the rate of publication**	Academic Dutch REC, Netherlands	1997–2006	2006	RCTs 49/100 (49%)	65% non profit, 34% profit, 1% missing information	We identified three prognostic indicators (REC letters, therapeutic benefit, and participant burden) of publication rate. After suitable replication, RECs might explore using prognostic indicators, such as these, to target study protocols at high risk for nonpublication. Discussing the risk of nonpublication with investigators could help prevent nonpublication (71% in this cohort).
Blumle 2008 [31]	**Publication rate: To estimate the publication rate overall and by study design, to identify factors associated with the full publication of study results and to determine the frequency of citation of corresponding articles.**	University of Freiburg REC, Germany	2000	2007	Included: RCTs 141/299 (47%) Completed : RCTs: 103/225 (46%)	Completed studies: 53% Commercial, 16% non commercial, 31% not stated	Results of German clinical research projects conducted are largely underreported. Barriers tosuccessful publication need to be identified and appropriate measures taken. Close monitoring of projects until publication and adequate support provided to investigators may help remedy the prevailing underreporting of research.
Hall 2007 [32]	**Study publication bias and time lag bias: we hypothesized that there would be less publication bias, and that the publication record of completed clinical trials from a single Canadian centre would be higher than previously reported.**	Capital District Health Authority Research Ethics Board (REB) in Halifax, Nova Scotia, Canada	1995–1996	2006	RCTs: 89/185 (48%)	68% Industry, 22% local health authority, 6% federal granting agency, 2% charity, 2% other^5^	Publication bias continues to be a problem, particularly for early phase investigative studies. Our results suggest that a different approach is required to reduce publication bias. The role that REBs and peer-reviewed journals might play requires further exploration.

1 Easterbrook et al assumed that only studies that had been analysed had the potential for being written up and published, so tests for study publication bias were restricted to these.

2 Studies for which there was a full interview by the researchers of the cohort study and for which information on the nature of results and publication was provided.

3 Of the 520 studies with completed questionnaires, 321 had analysis undertaken with results available and were included in further analysis of the association between study outcome and time to publication.

4 Both groups are not mutually exclusive. 4% had a statement of both sources of funding. In the remainder, the protocols did not include information on how study was funded.

5 This includes 5 studies not approved but it is unclear where the funding from these 5 studies was from.

NI No information available.

#### Study publication bias

Four of the empirical studies investigating study publication bias also assessed time lag bias [4], [5], [7], [32], four [21], [27], [28], [31] assessed the outcome of protocols submitted to a research ethics committee (for example whether trials were started and if they were published) and another considered whether absence of acknowledged funding hampered implementation or publication [22]. Eleven of the empirical studies [4], [7], [19], [21]–[24], [27], [28], [31], [32] assessed protocols approved by ethics committees, one [3] assessed those approved by health institutes, one assessed trials processed through a hospital pharmacy [30], one assessed studies funded by the NHS and commissioned by the North Thames Regional Office [20] and one empirical study [5] assessed trials conducted by NIH-funded clinical trials groups. The time period between protocol approval and assessment of publication status varied widely (less than one year to 34 years).

#### Outcome reporting bias

Four of the empirical studies [17], [25], [26], [29] assessed protocols approved by ethics committees and one empirical study [18] assessed those approved by a health institute. The time period between protocol approval and assessment of publication status varied from four to eight years.

### Quality Assessment

Details of the methodological quality are presented in Table 2. The overall methodological quality of included empirical studies was good, with more than half of studies meeting all criteria.

**Table 2:** Methodological Quality Assessment.

Quality criteria	Inception cohort	Complete follow up of all trials	Publication ascertained through personal contact with investigators	Definition of positive and negative findings clearly defined	Comparison of protocol to publication
Easterbrook, 1991 [24]	Y	N (25% lost to follow up)	Y	Y (positive: p<0.05/striking, negative: p≥0.05/definite but not striking, null: no difference observed between the groups/null findings.)	NA
Dickersin, 1992 [23]	Y	Y	Y	Y (positive: p<0.05/statistically significant, negative: suggestive trend but not statistically significant, null: no trend or difference. In terms of importance when statistical tests were not performed: great, moderate or little.)	NA
Dickersin, 1993 [3]	Y	N (14% refused to participate)	Y	Y (positive: p<0.05 significant/of great importance, negative: showing a trend in either direction but not statistically significant/moderate importance/no difference/little importance.),	NA
Stern, 1997 [4]	Y	N (only 70% of questionnaires were completed)	Y	Y (positive: p<0.05 significant/striking/important/definite, negative: non-significant trend 0.05≤p<0.10 or non-significant or null p≥0.10/unimportant and negative)	NA
Cooper, 1997 [19]	Y	Y	Y	N (significant and non-significant)	NA
Wormald, 1997 [30]	Y	Y	Y	Y (positive: p<0.05, negative: p≥0.05)	NA
Ioannidis, 1998 [5]	Y	Y	Y	Y (positive: p<0.05 significant and in favour of experimental therapy arm or any arm when there is no distinct control, negative: nonstatistically significant findings or favouring the control arm )	NA
Pich, 2003 [27]	Y	Y	Y	NA	NA
Cronin, 2004 [20]	Y	Y	Y	U (study showed effect)	NA
Decullier, 2005 [7]	Y	N (only 69% of questionnaires were completed)	Y	Y (confirmatory/inconclusive/invalidating)	NA
Decullier, 2006 [22]	Y	N (only 80% of questionnaires were completed)	Y	Y (scale from 1 to 10 for not important to very important)	NA
Hahn, 2002 [26]	Y	Y	Y	NA	Y
Chan, 2004a [18]	Y	Y	Y	Y (positive: p<0.05, negative: p≥0.05)	Y
Chan, 2004b [17]	Y	Y	Y	Y (positive: p<0.05, negative: p≥0.05)	Y
Ghersi, 2006 [25]	Y	Y	Y	Y (positive: p≤0.05, negative: p>0.05)	Y
Von Elm, 2008 [29]	Y	Y (for drug trials)	Y	Y (positive: p<0.05)	Y
Turer 2007 [28]	Y	Y (unable to contact trialists in 9%)	Y	NA	NA
De jong 2010 [21]	Y	N (20% did not respond to questionnaire)	Y	NA	NA
Blumle 2008 [31]	Y	Y (87% response overall, unclear on RCTs)	Y	NA	Y but data not yet published
Hall 2007 [32]	Y	Y	Y	Y statistically significant findings (i.e., major outcome variable under study reported to be statistically different from comparator at *P*<0.05 level)	NA

Y yes.

N no.

U unclear.

NA Not applicable.

#### Study publication bias

Seven of the fifteen empirical studies [5], [7], [27], [28], [30]–[32] met all four of the criteria for studies investigating study publication bias (inception cohort, complete follow up of all trials, publication ascertained through personal contact with the investigator and definition of positive and negative findings clearly defined). In six empirical studies [3], [4], [7], [21], [22], [24] there was less than 90% follow up of trials and in two empirical studies [19], [20] the definition of positive and negative findings was unclear.

#### Outcome reporting bias

All five empirical studies [17], [18], [25], [26], [29] met all five criteria for studies investigating ORB (inception cohort, complete follow up of all trials, publication ascertained through personal contact with the investigator, definition of positive and negative findings clearly defined and comparison of protocol to publication).

As some studies may have several specified primary outcomes and others none, we looked at how each of the empirical studies dealt with this: Hahn et al [26] looked at the consistency between protocols and published reports in regard to the primary outcome and it was only stated that there were two primary outcomes in one study. In both of their empirical studies Chan et al [17], [18] distinguished harm and efficacy outcomes but did consider the consistency of primary outcomes between protocols and publications and stated how many had more than one primary outcome. Ghersi et al [25] included studies with more than one primary outcome and included all primary outcomes in the analysis but excluded studies with primary outcomes that were non identifiable or included more than two time points. This is due to complex outcomes being more prone to selective reporting. von Elm et al [29] considered harm and efficacy outcomes and primary outcomes.

### Flow Diagrams

The flow diagrams (Figures 3, 4, 5, 6, 7, 8, 9, 10, 11, 12, 13, 14, 15, 16, 17, 18, 19, 20, 21, 22) show the status of approved protocols in included empirical studies based on available publications and additional information obtained such as number of studies stopped early or never started.

**Figure 3:**
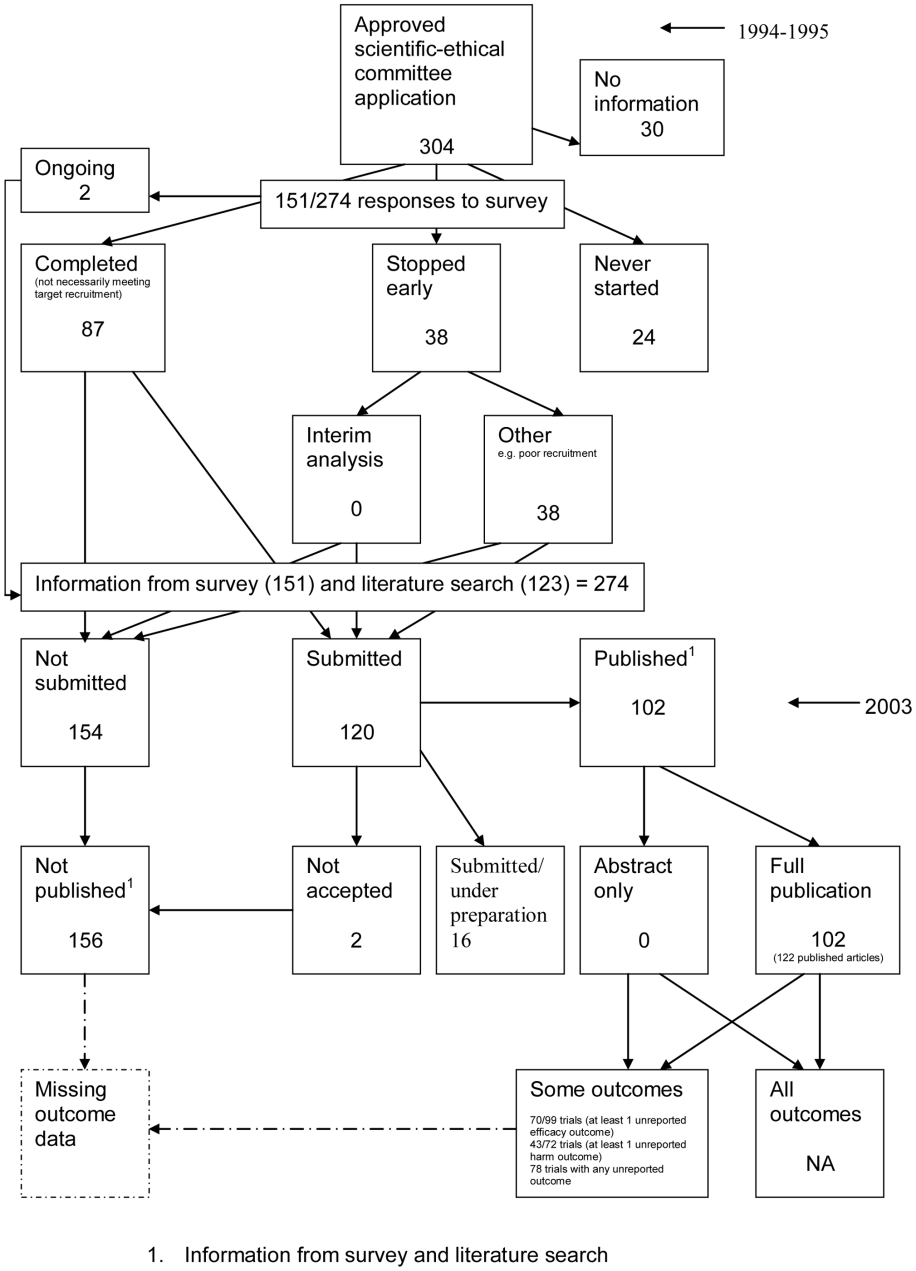
Status of approved protocols for Chan 2004b study [17].

**Figure 4:**
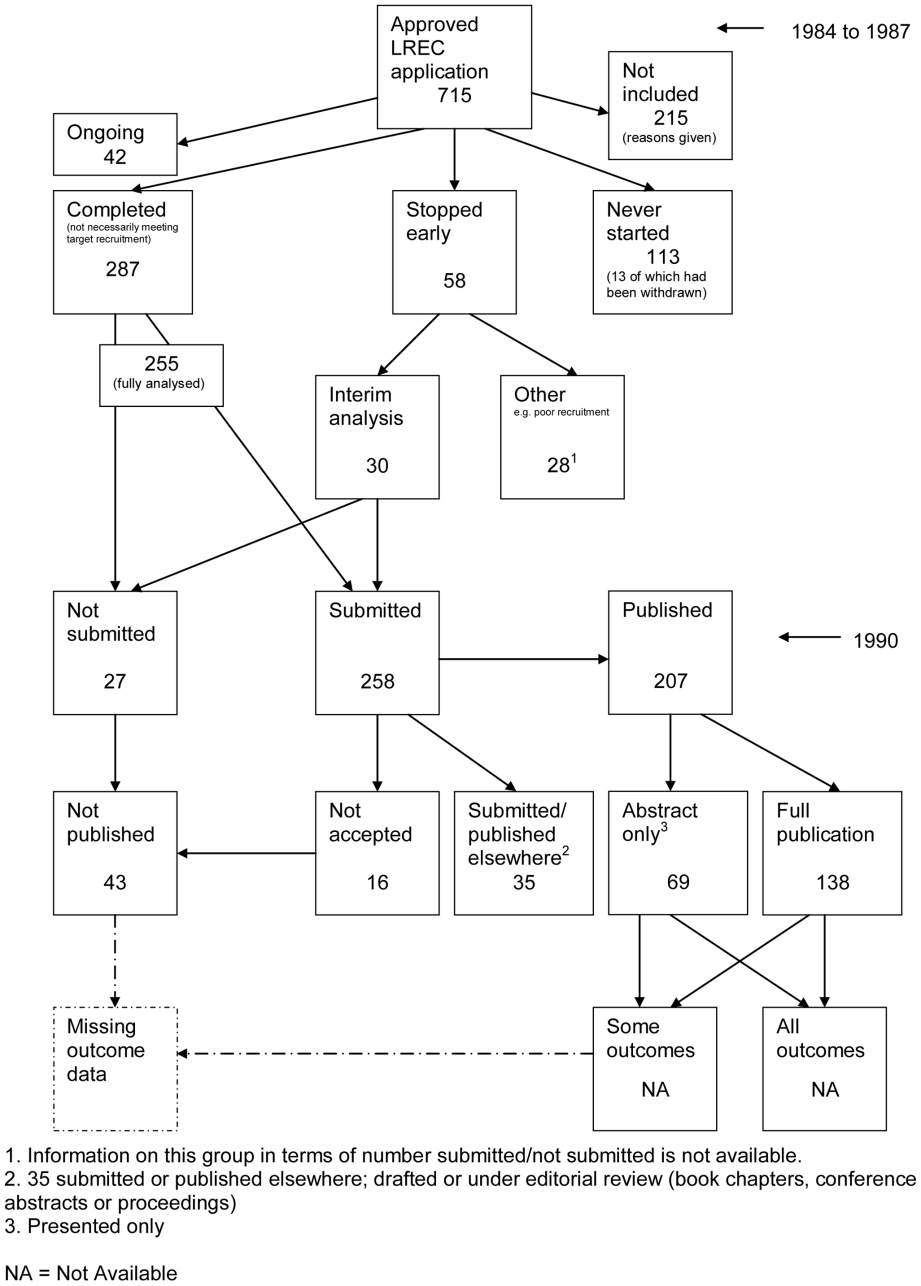
Status of approved protocols for Easterbrook 1991 study [24].

**Figure 5:**
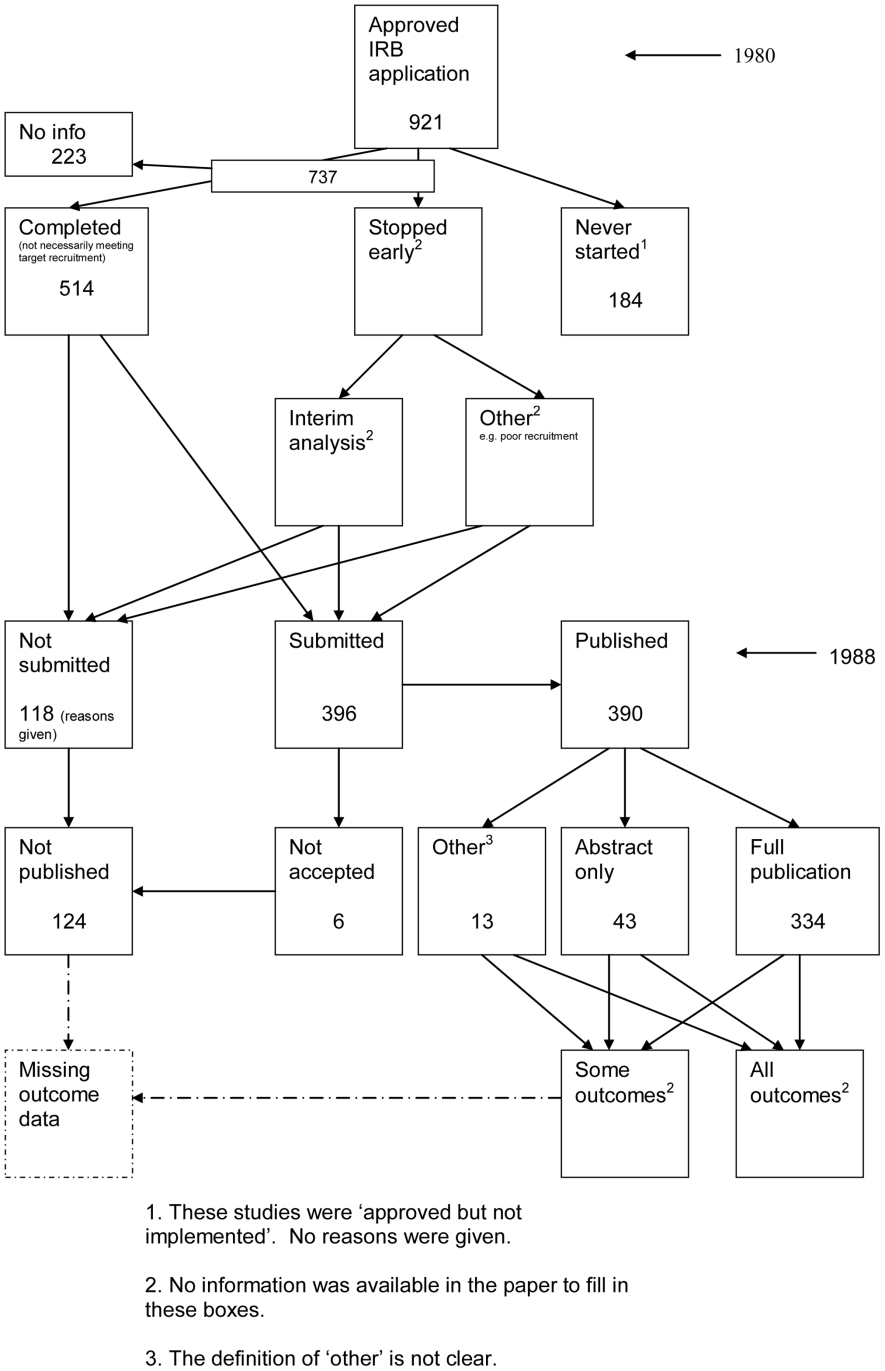
Status of approved protocols for Dickersin 1992 study [23].

**Figure 6:**
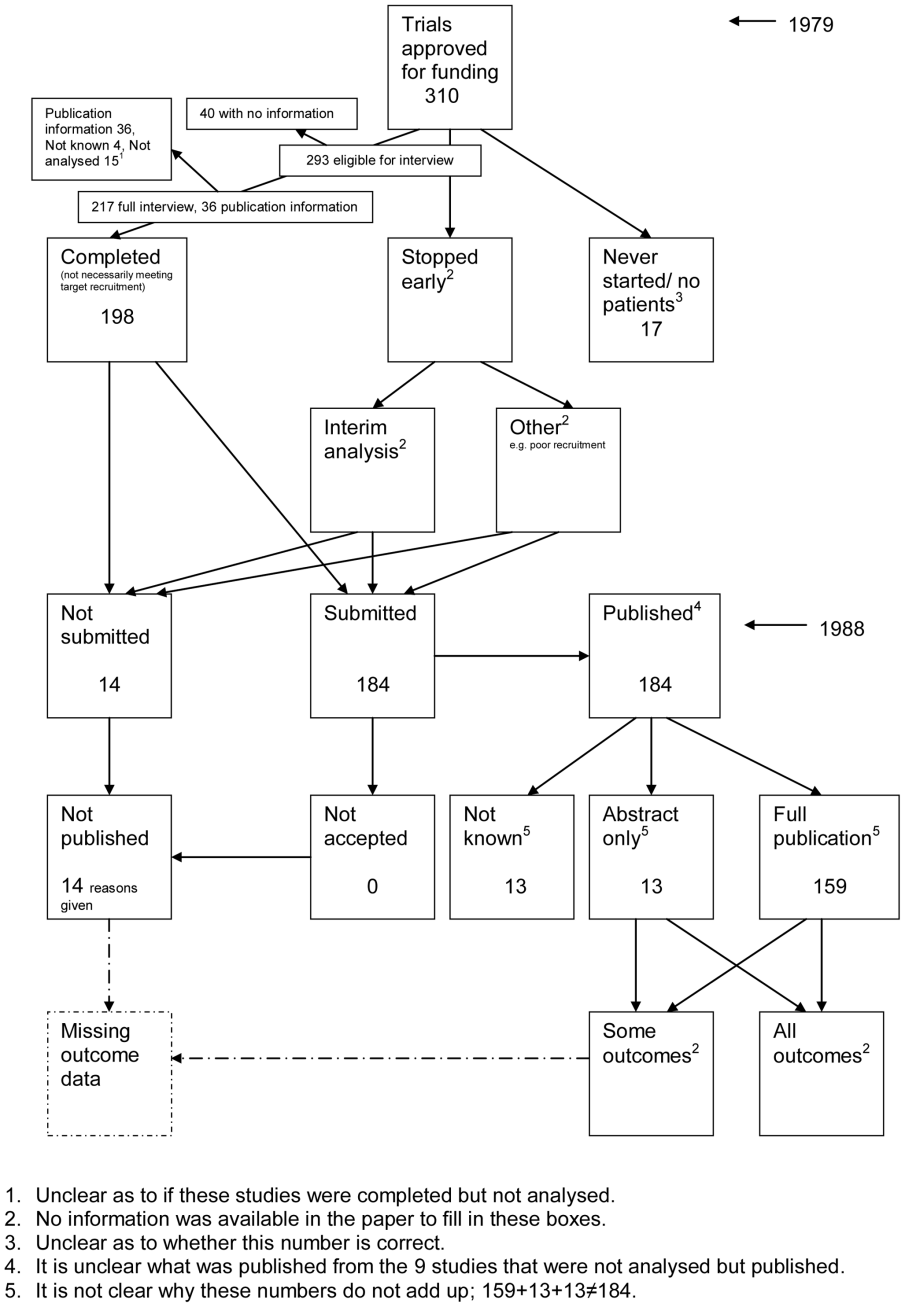
Status of approved protocols for Dickersin 1993 study [3].

**Figure 7:**
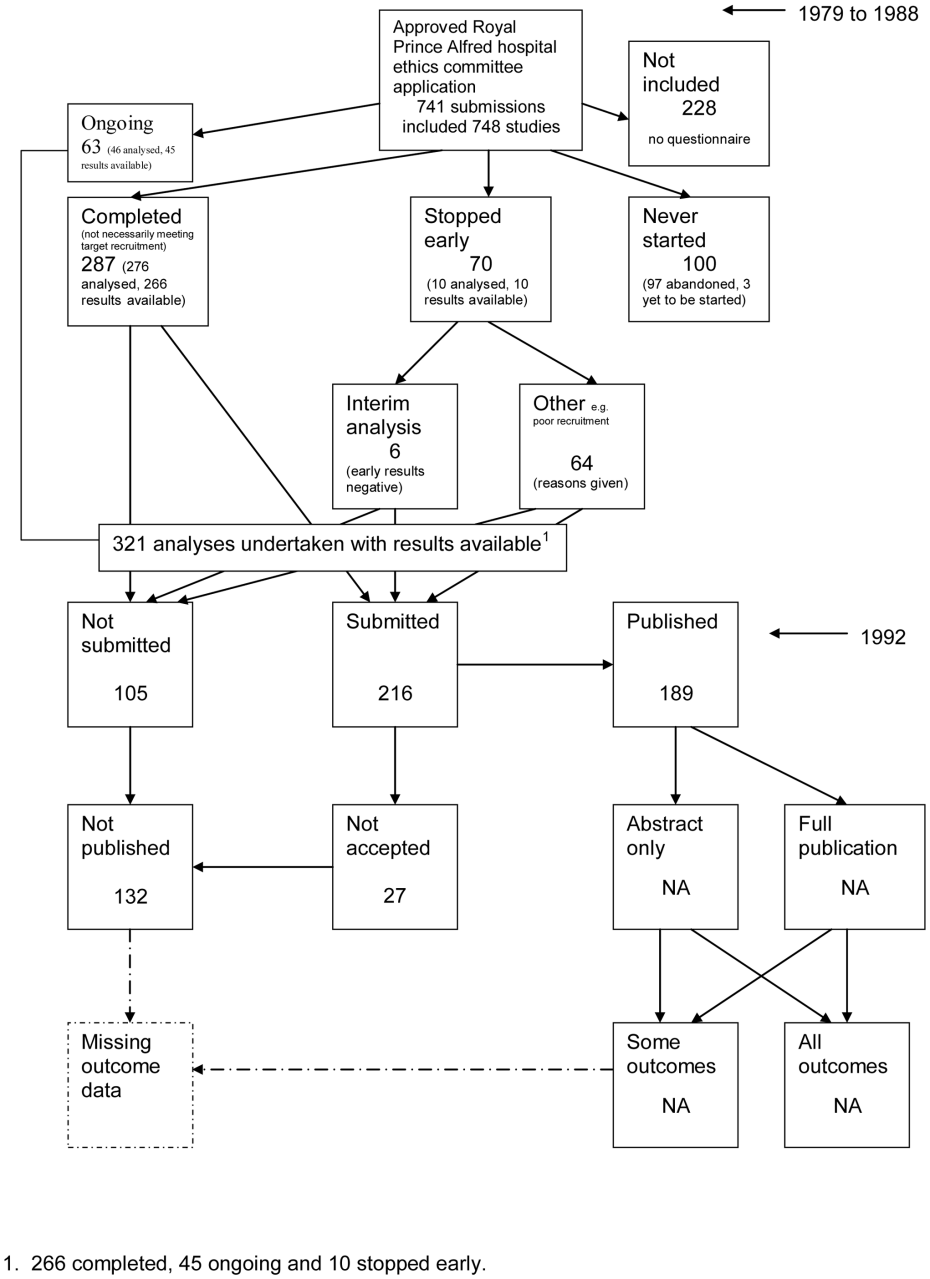
Status of approved protocols for Stern 1997 study [4].

**Figure 8:**
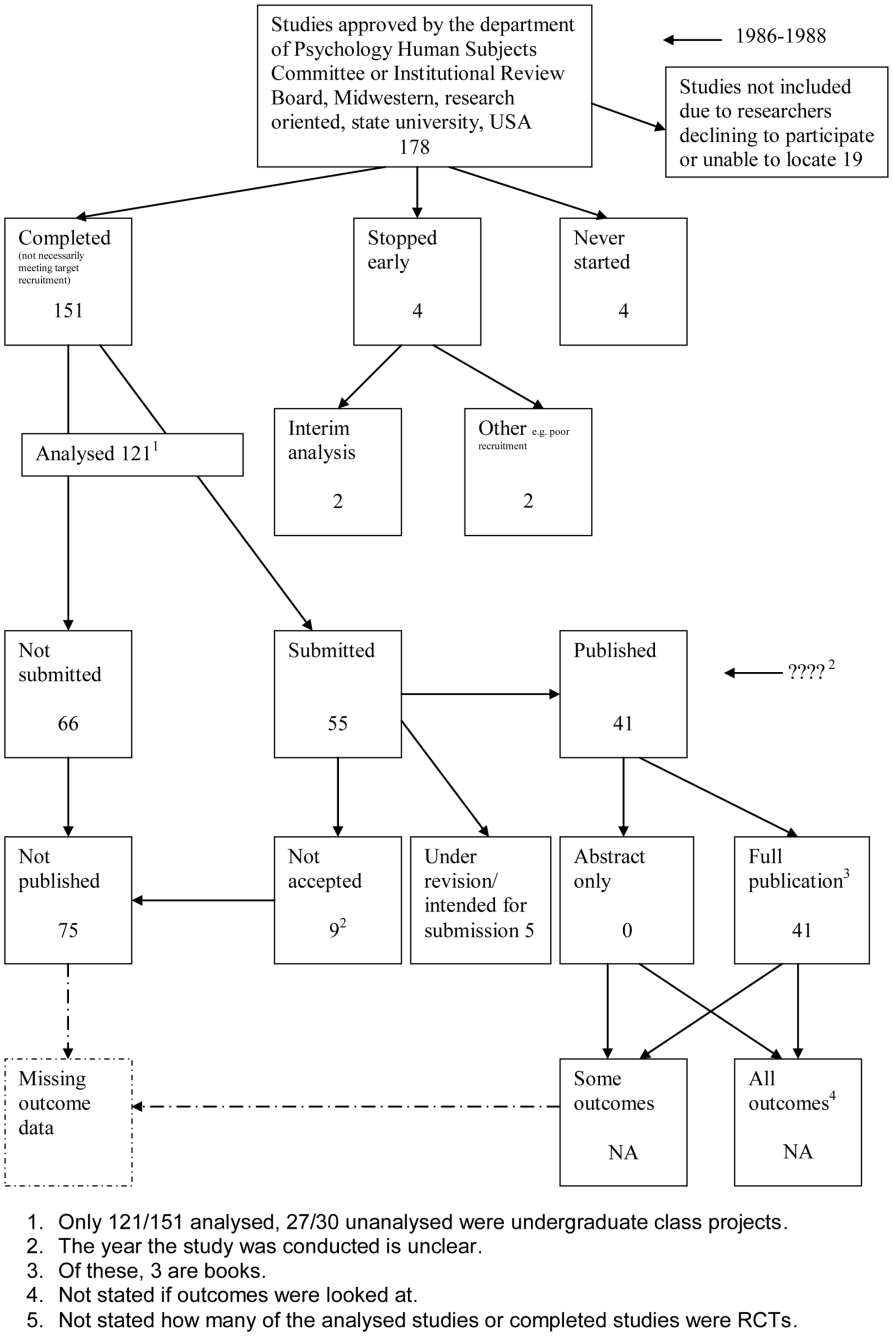
Status of approved protocols for Cooper 1997 study [19].

**Figure 9:**
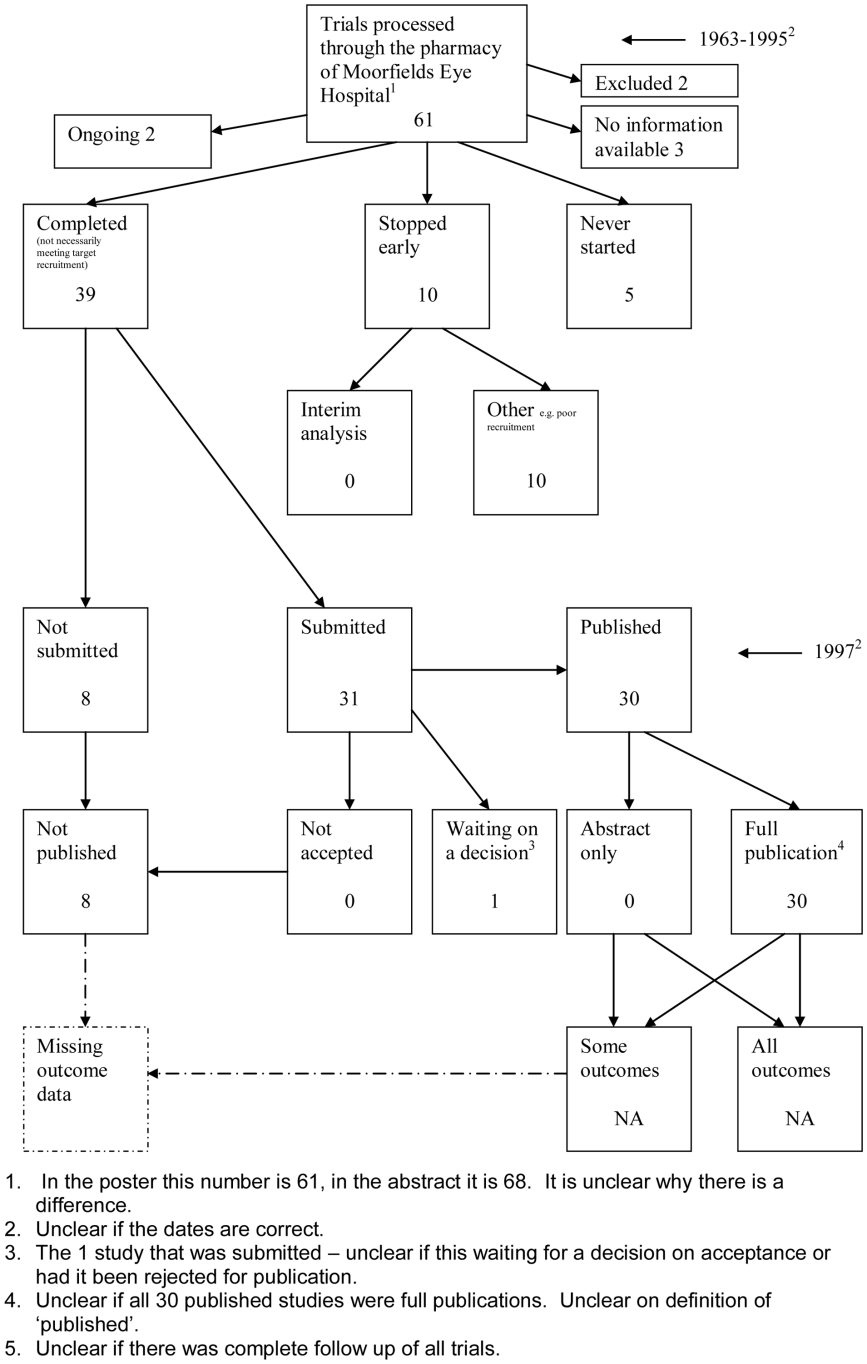
Status of trials for Wormald 1997 study [30].

**Figure 10:**
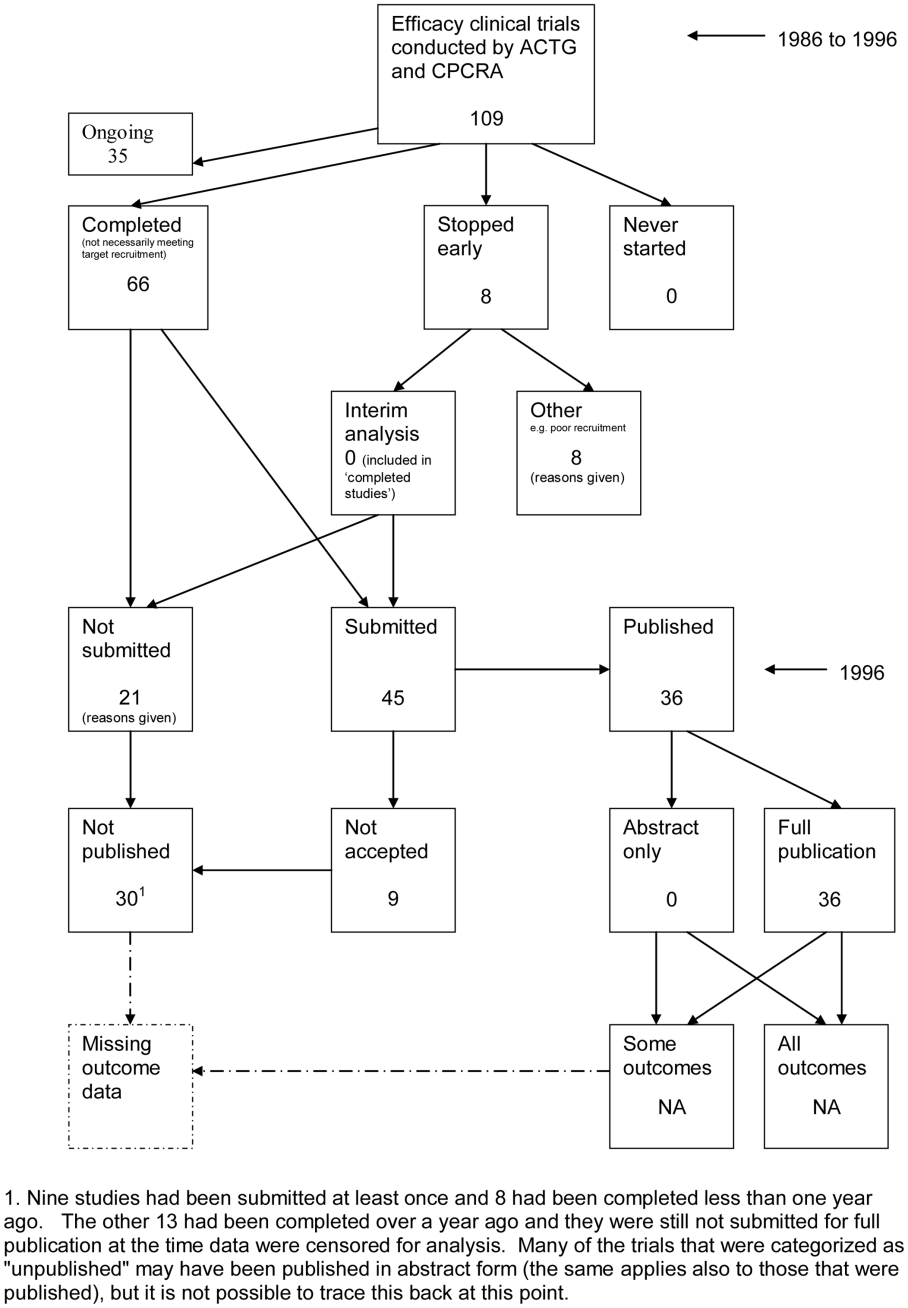
Status of approved protocols for Ioannidis 1998 study [5].

**Figure 11:**
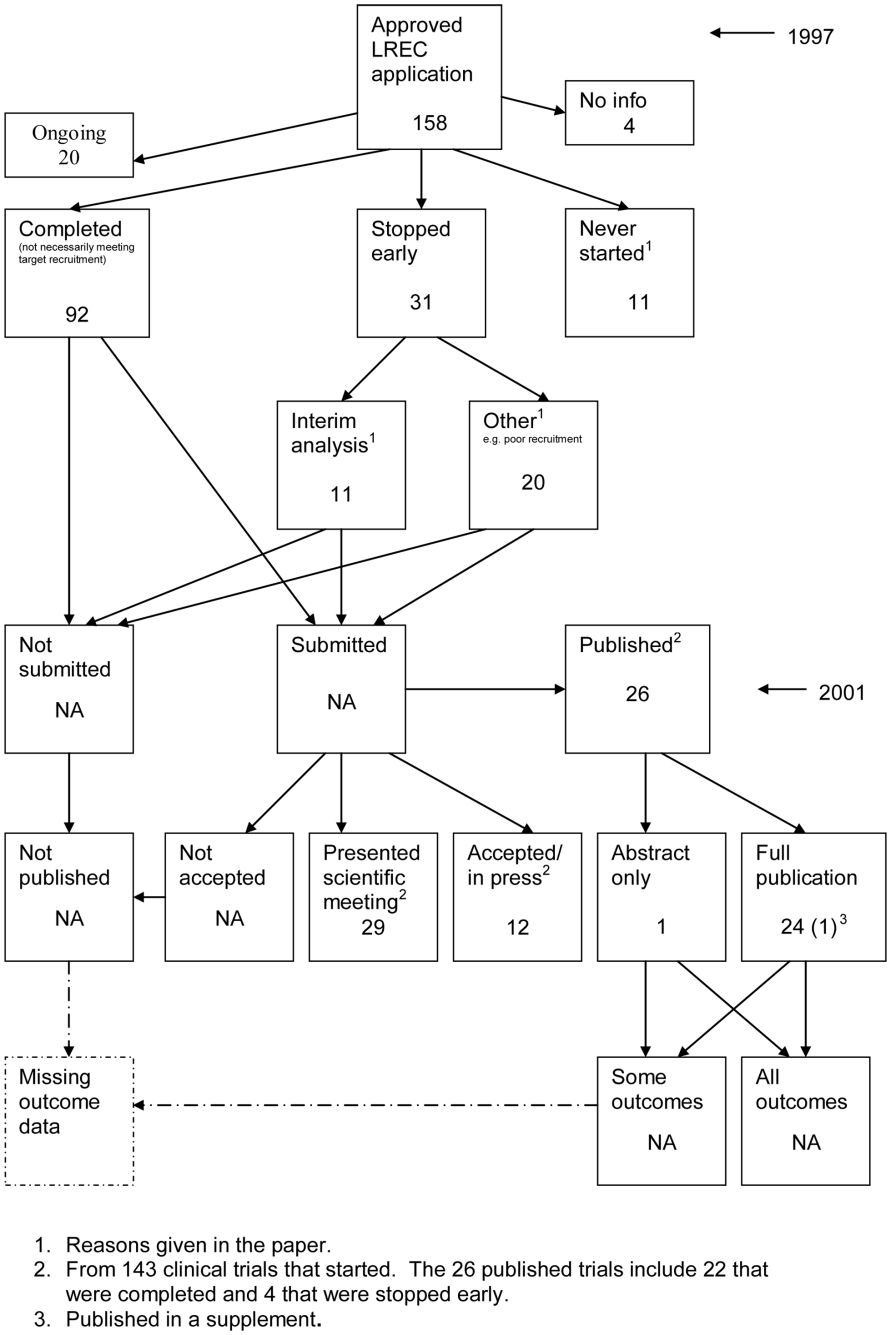
Status of approved protocols for Pich 2003 study [27].

**Figure 12:**
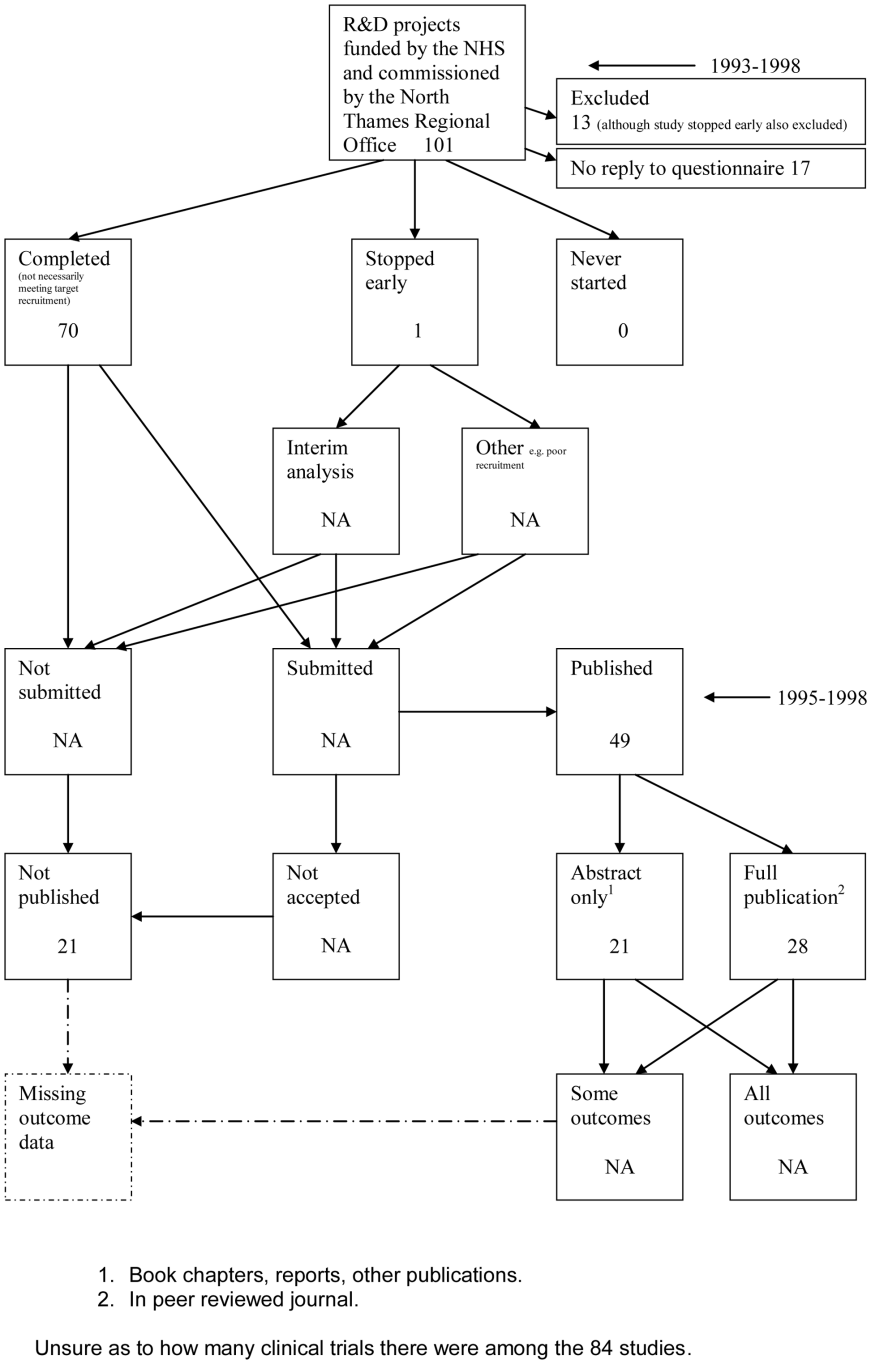
Status of approved protocols for Cronin 2004 study [20].

**Figure 13:**
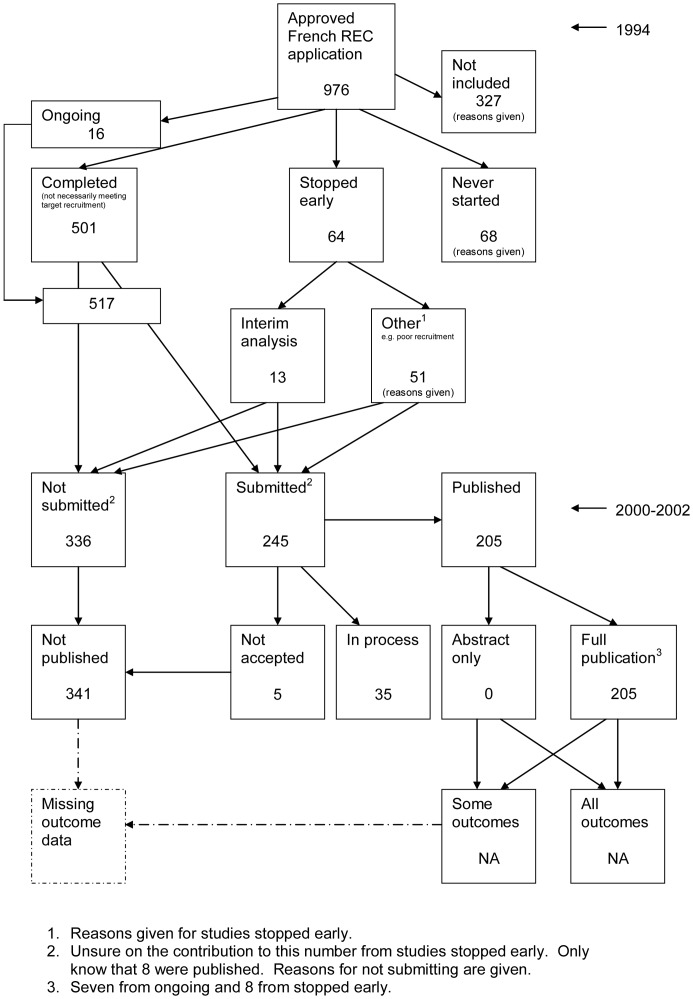
Status of approved protocols for Decullier 2005 study [7].

**Figure 14:**
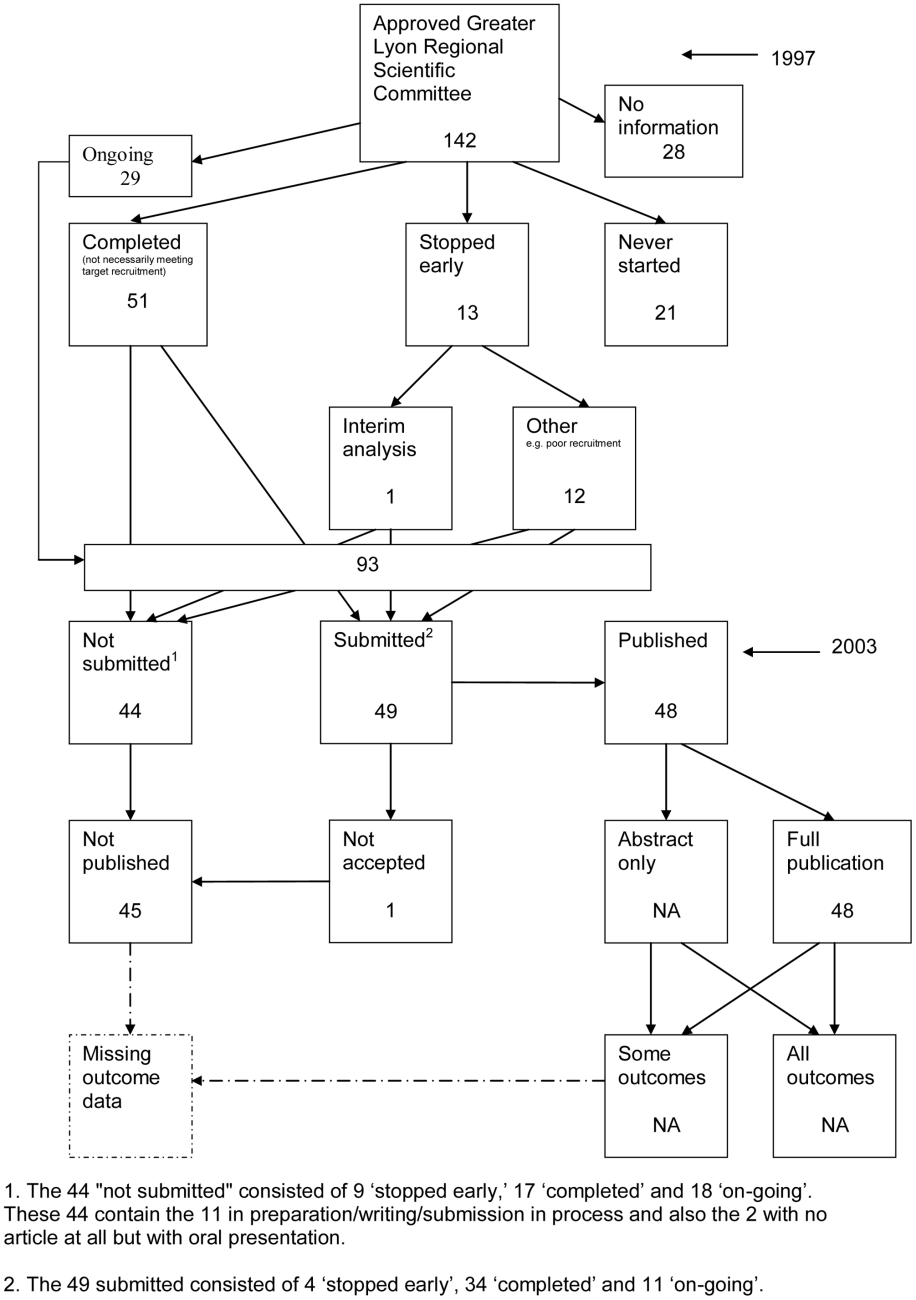
Status of approved protocols for Decullier 2006 study [22].

**Figure 15:**
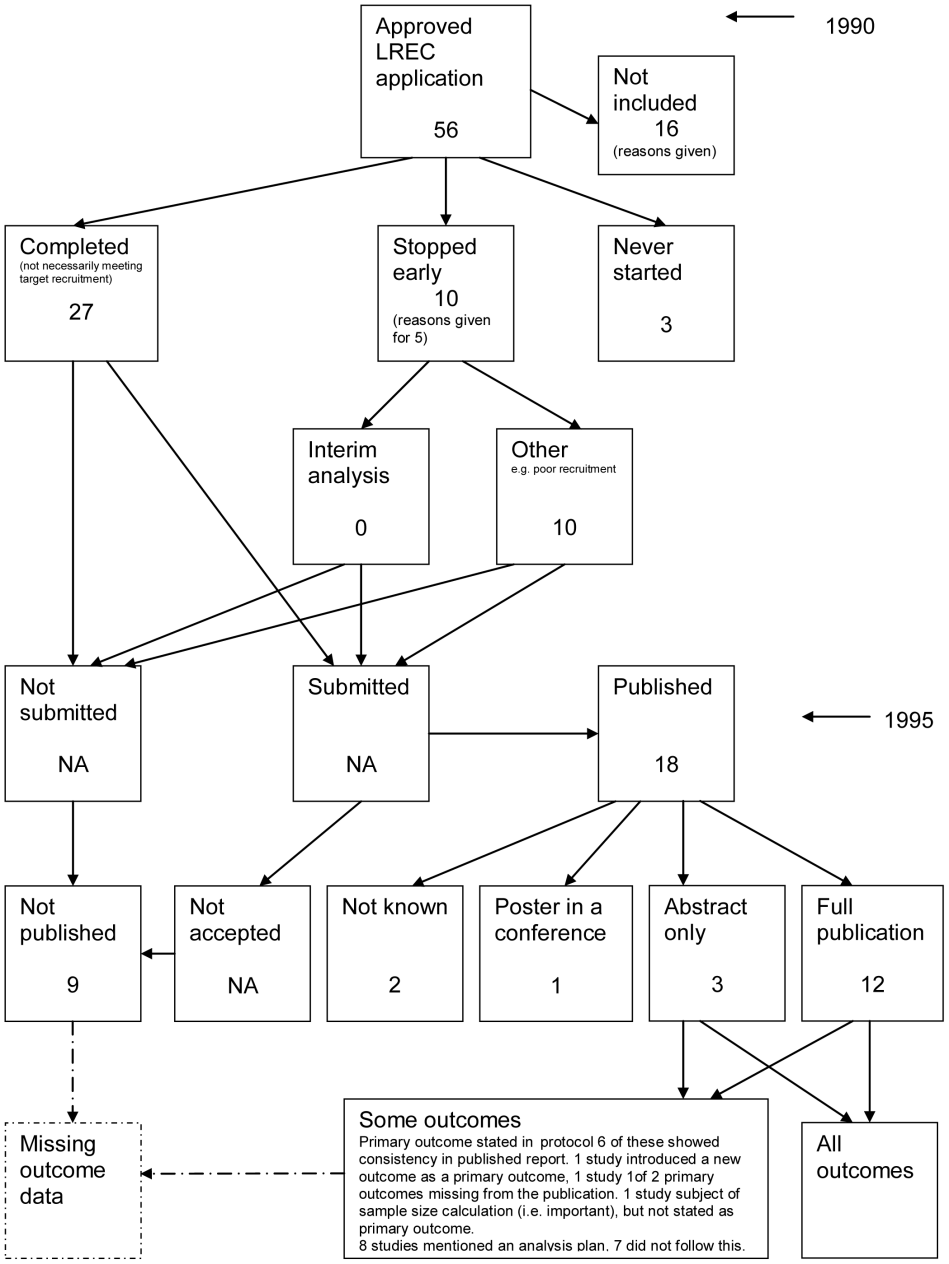
Status of approved protocols for Hahn 2002 study [26].

**Figure 16:**
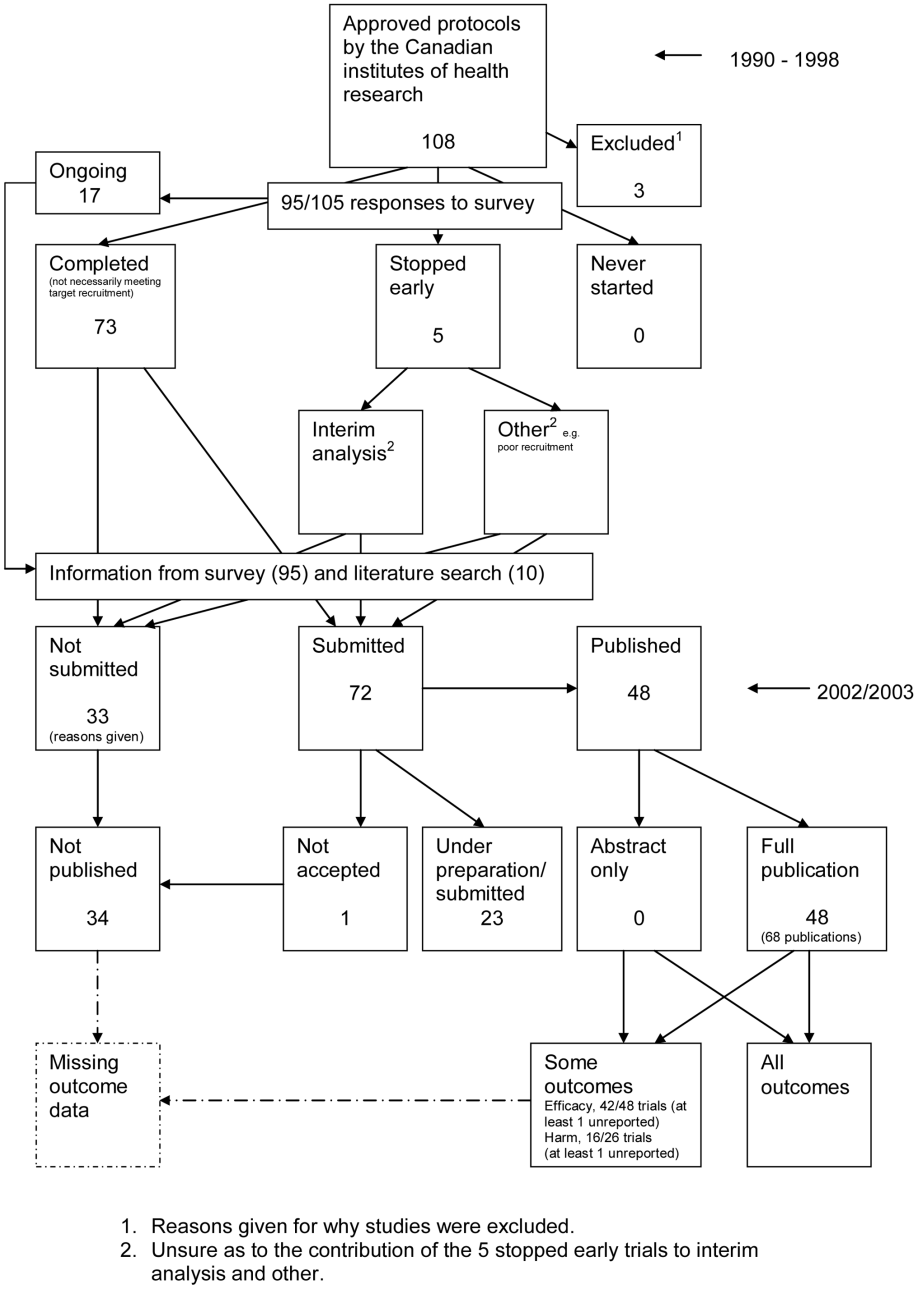
Status of approved protocols for Chan 2004a study [18].

**Figure 17:**
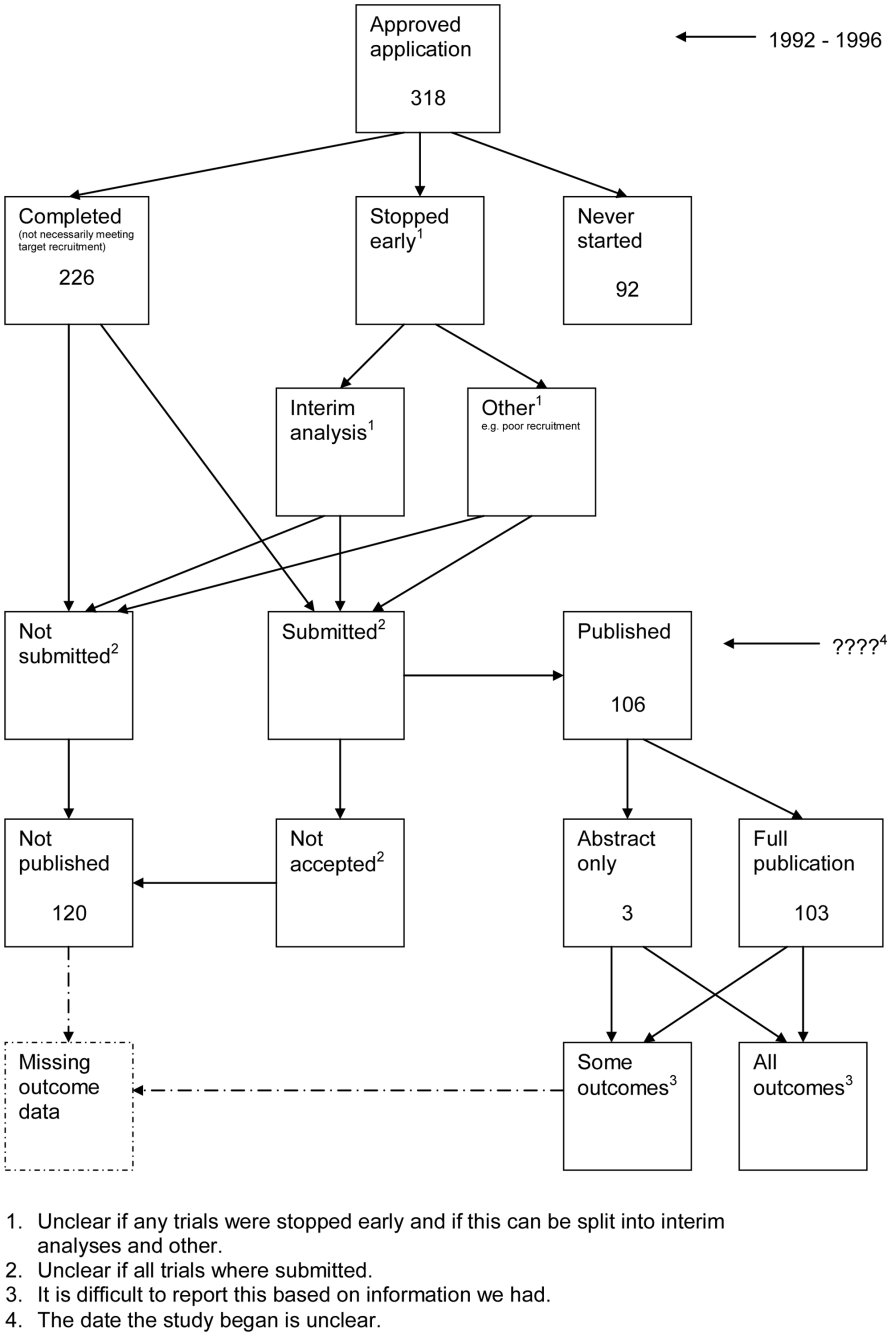
Status of approved protocols for Ghersi 2006 study [25].

**Figure 18:**
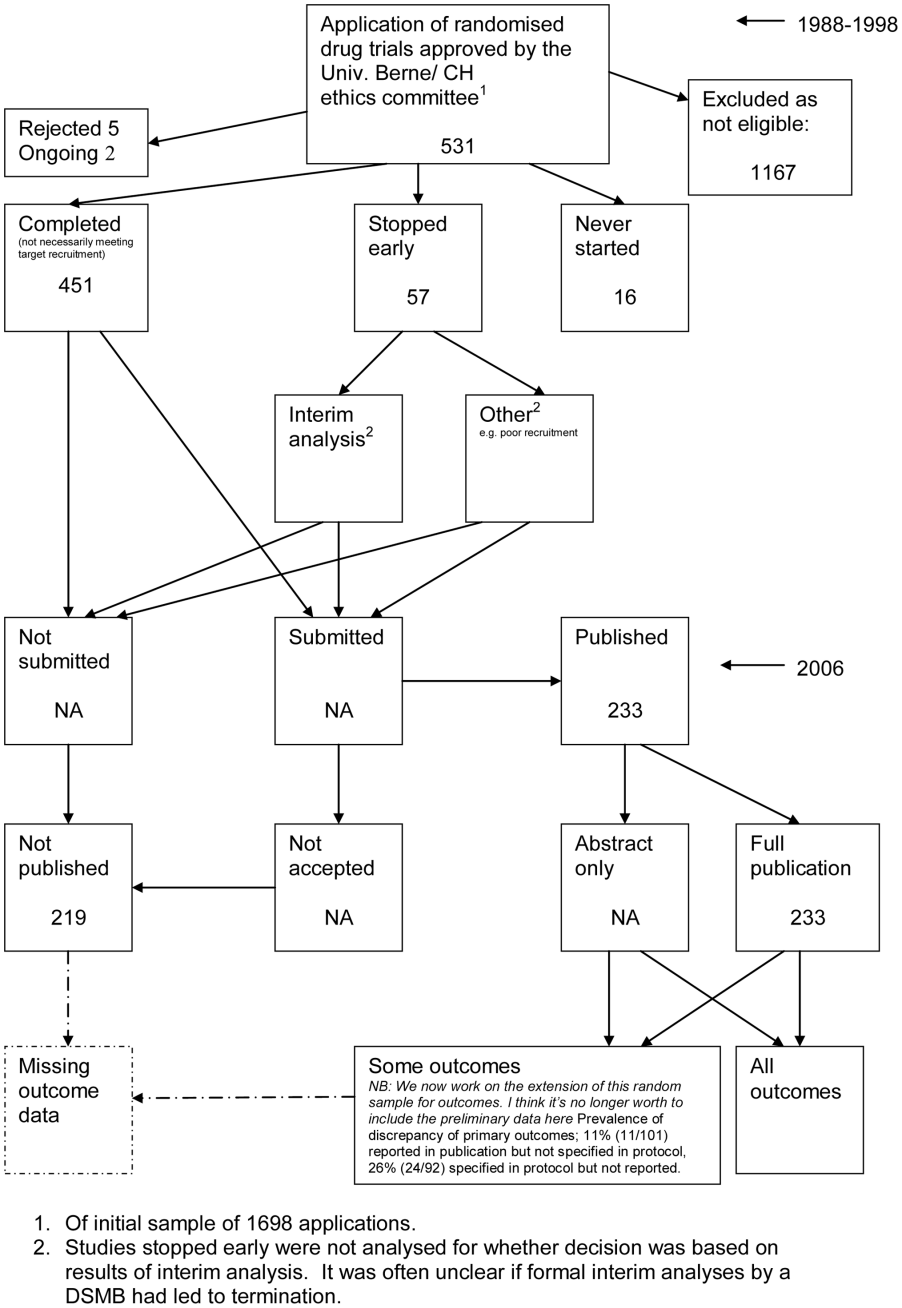
Status of approved protocols for von Elm 2008 study [29].

**Figure 19:**
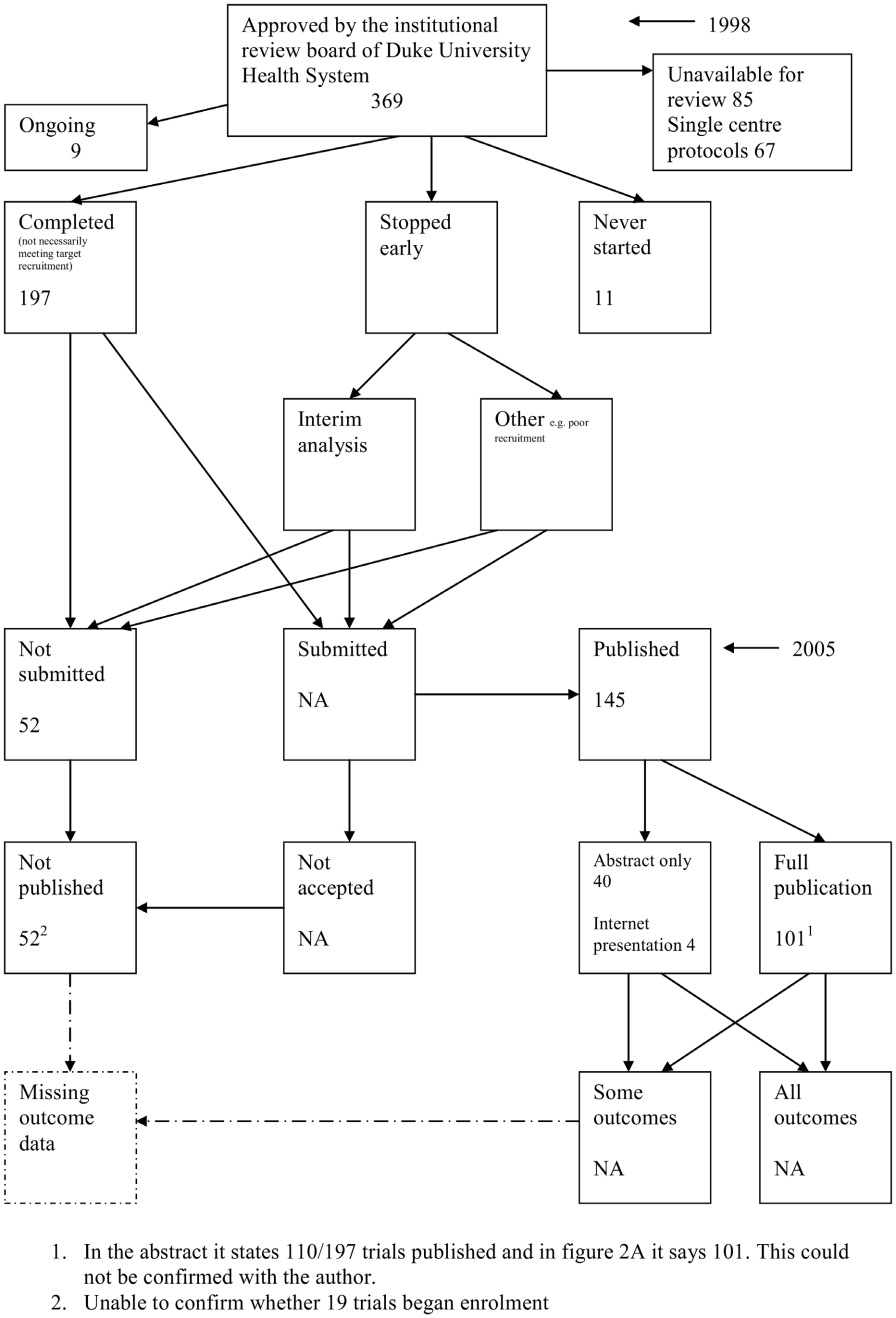
Status of approved protocols for Turer 2007 study [28].

**Figure 20:**
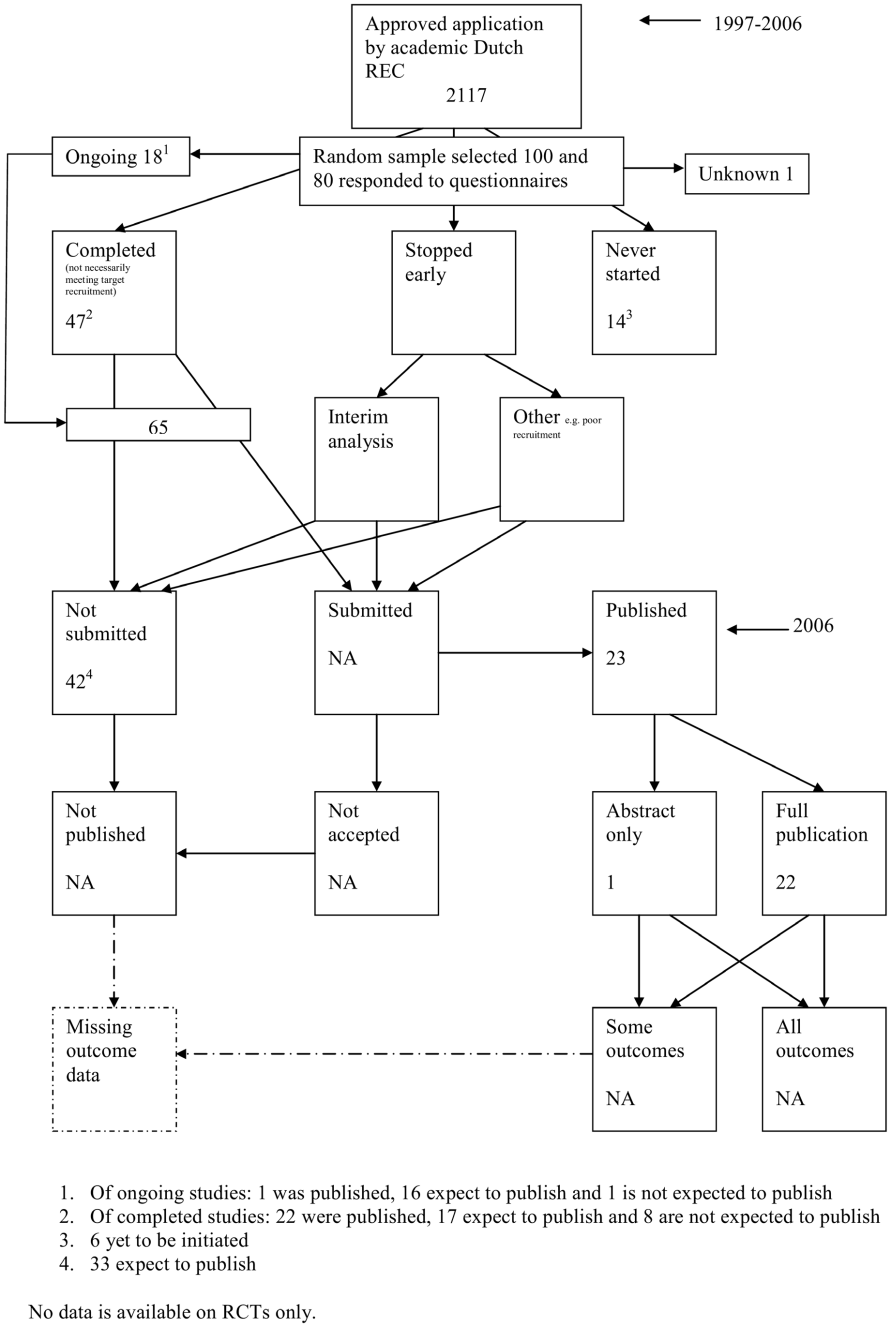
Status of approved protocols for De Jong 2010 study [21].

**Figure 21:**
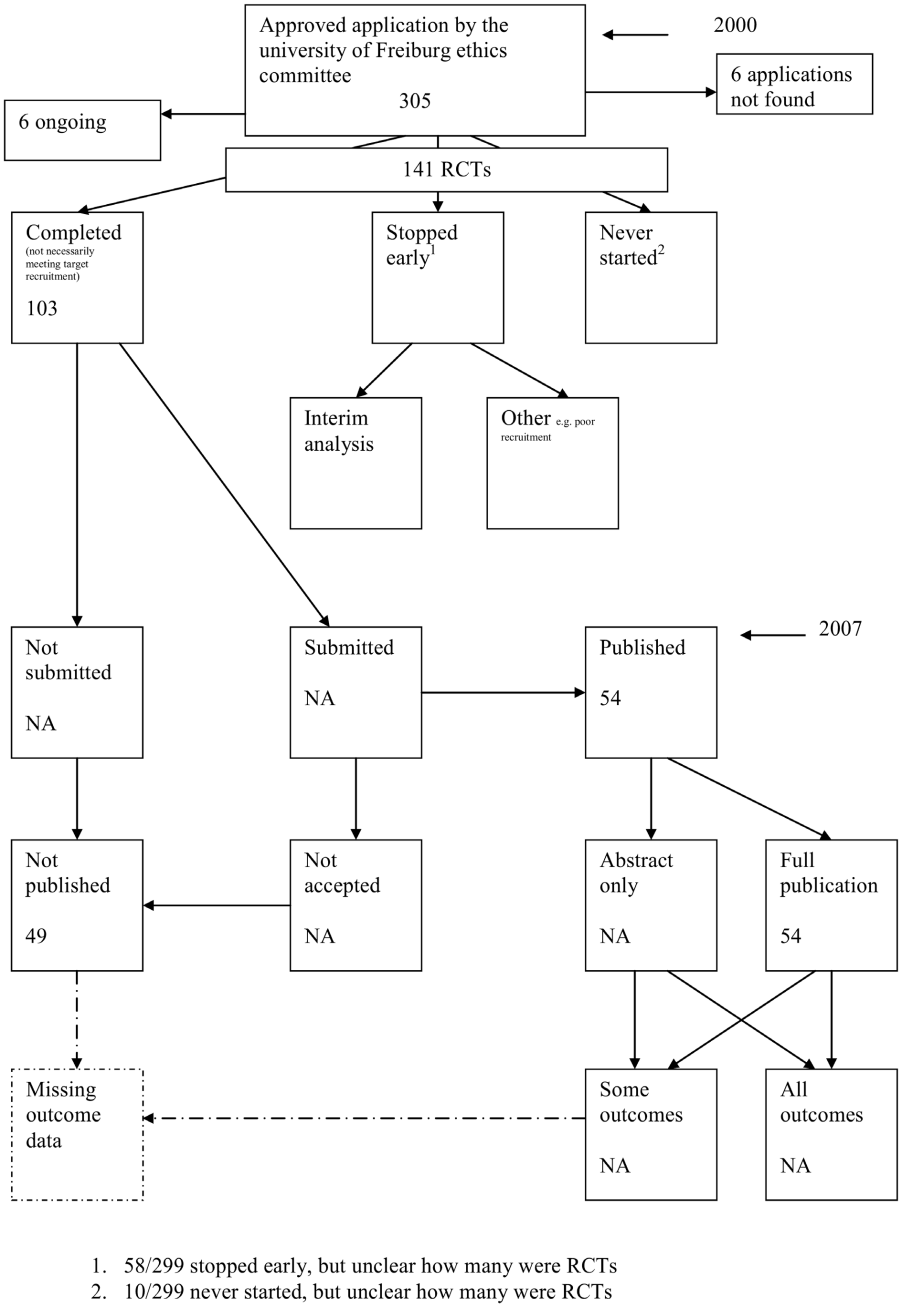
Status of approved protocols for Blumle 2008 study [31].

**Figure 22:**
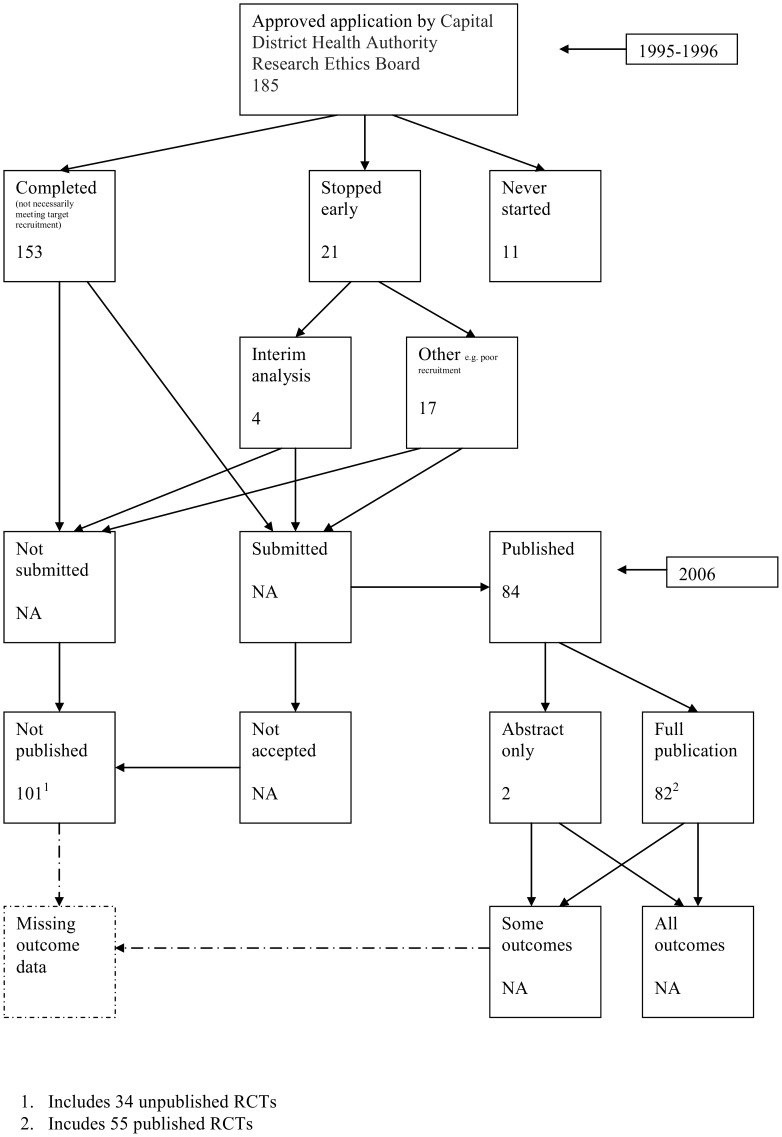
Status of approved protocols for Hall 2007 study [32].

#### Study publication bias

No information other than the study report was available for one empirical study [24] due to its age. Information could not be located for four empirical studies [3], [19], [23], [28]. A conference abstract and poster was only available for one empirical study presented over 10 years ago [30]. Extra information from lead or contact authors was available for nine empirical studies [4], [5], [7], [20]–[22], [27], [31], [32], including data to complete flow diagrams, information on definitions and clarifications.

#### Outcome reporting bias

Extra information from lead or contact authors was available for four empirical studies [17], [18], [26], [29], including data to complete flow diagrams, information on definitions, clarifications and extra information on outcomes. Original flow diagrams and questions asked are available on request.

Figure 3 shows for illustrative purposes the completed flow diagram for the empirical study conducted by Chan et al [17] on the status of 304 protocols approved by the Scientific-Ethical Committees for Copenhagen and Frederiksberg in 1994–1995. The empirical study was conducted in 2003, which allowed sufficient time for trial completion and publication. Thirty studies were excluded as the files were not found. Surveys were sent to trial investigators with a response rate of 151 out of 274 (55%); of these two were ongoing, 38 had stopped early, 24 studies had never started and 87 studies were completed. Information from the survey responses (151) and the literature search alone (123) indicated that 120 studies had been submitted for publication and 154 studies had not been submitted for publication. Of the 120 submitted studies; 102 had been fully published, 16 had been submitted or were under preparation and two had not been accepted for publication. This resulted in 156 studies not being published.

### Publication and Trial Findings

#### Study publication bias

Table 3 shows the total number of studies published in each cohort which varies widely from 21% to 93%. Nine of the cohorts [3]–[5], [7], [19], [22]–[24], [30] consider what proportion of trials with positive and negative results are published, ranging from 60% to 98% and from 19% to 85%, respectively. Only four cohorts [4], [7], [19], [24] consider what percentage of studies with null results (no difference observed between the two study groups, *p*>0.10, inconclusive) are published (32% to 44%). The results consistently show that positive studies are more likely to be published compared to negative studies.

**Table 3:** Publication and trial findings.

Study ID	Total published (percentage)	Positive (percentage)	Negative (percentage)	Null (percentage)
Easterbrook, 1991 [24]	138/285 (48%)	93/154 (60%)	12/34 (35%)	33/97 (34%)
Dickersin, 1992 [23]	390/514 (76%)	260/314 (83%)	130/200 (65%)	NI
Dickersin, 1993 [3]	184/198 (93%)	121/124 (98%)	63/74 (85%)	NI
Stern, 1997 [4]	189/321 (59%)	153/232 (66%)	13/37 (35%)	23/52 (44%)
Cooper, 1997 [19]	38/121 (status known for 117/121) (31%)	-	-	-
Wormald, [30]	30/61 (status known for 39 completed trials) (49%)	14/15 (93%)	15/21 (71%)	NI
Ioannidis, 1998 [5]	36/66 (55%)	20/27 (74%)	16/39 (41%)	NI
Pich, 2003 [27]	26/123 (21%)	NI	NI	NI
Cronin, 2004 [20]	28/70 (40%)	NI	NI	NI
Decullier, 2005 [7]	205/649 (32%) (status known for 248^1^)	129/188 (67%)	3/16 (19%)	14/44 (32%)
Decullier, 2006 [22]	48/93 (status known for 47/51 completed trials) (52%)	26/37 (70%)	6/10 (60%)	NI
Hahn, 2002 [26]	18/27 (67%)	NI	NI	NI
Chan, 2004a [18]	48/105 (46%)	NI	NI	NI
Chan, 2004b [17]	102/274 (37%)	NI	NI	NI
Ghersi, 2006 [25]	103/226 (46%)	NI	NI	NI
Von Elm, 2008 [29]	233/451 (52%)	NI	NI	NI
Turer 2007 [28]	101/217 (47%)	NI	NI	NI
De jong 2010 [21]	22/80 (28%)	NI	NI	NI
Blumle 2008 [31]	RCTs: 54/103 (52%); Overall: 109/225 (48%)	NI	NI	NI
Hall 2007 [32]	RCTs: 55/89 (62%); Overall: 84/185 (45%)	71/84 (85%)	13/84 (15%)	NI

1. Analysis restricted to 248 completed, non confidential, with hypothesis tests and direction of results.

NI No information, this study does not look at this.

− Not able to work out values.

Status implies positive or negative findings.

Table 4 shows general consistency in the definition of ‘published.’ However, two empirical studies [3], [23] considered grey literature in their definition of ‘published’ although information on full publications and grey literature publications are separated (Figures 5, 6). Although not considered in the definition of ‘published’, seven empirical studies [7], [21], [22], [24], [27], [28], [32] gave information on the grey literature or reports in preparation. Three empirical studies gave no information on their definition of ‘published’ [19], [20], [30]. In addition, results are presented for the percentage of studies not submitted for journal publication (7% to 58%), of studies submitted but not accepted for publication (0 to 20%) by the time of analysis of the cohort and the percentage of studies not published that were not submitted (63% to 100%). This implies that studies remain unpublished due largely to failure to submit rather than rejection by journals.

**Table 4:** Aspects of study level publication bias.

Study	Definition of published article employed by eligible studies	Time since protocol approved	% Not submitted^1^ (95% CI)	% Submitted but not accepted^2^ (95% CI)	% Studies not published that were not submitted (95% CI)
Easterbrook, 1991 [24]	Acceptance by a journal (published or in press), but not book chapters or published meeting abstracts or proceedings.	3 years	9 (6, 12)	6 (3, 9)	63 (49, 77)
Dickersin, 1992 [23]	Reported in one or more journal articles, monographs, books, or chapters in books, were available from medical libraries or were in documents available from a published archive, e.g., the National Technical Information service	8 years	23 (19, 27)	1.5 (0, 3)	95 (91, 99)
Dickersin, 1993 [3]	Abstracts, journal articles, book chapters, proceedings, letter to the editor, or other material.	9 years	7 (3, 11)	0	100
Stern, 1997^3^ [4]	As an article in a peer reviewed journal	4 plus years	33 (28, 38)	13 (8,17)	80 (73, 87)
Cooper, 1997 [19]	NI	NI	NI		
Wormald, 1997 [30]	NI	2 to 34 years	21 (8,34)	0	100
Ioannidis, 1998 [5]	Peer-reviewed journal articles	12% completed less than 1 year ago, 20% completed over 1 year ago.	32 (21, 43)	20 (8, 32)	70 (54, 86)
Pich, 2003 [27]	Peer reviewed journal articles	3 years	*		
Cronin, 2004 [20]	NI	1 to 5 years	*		
Decullier, 2005^3^ [7]	Published as a scientific paper. Grey literature – published in other formats than scientific papers, that is generally not accessible through libraries (internal reports, theses, abstracts, posters)	6 years (2% not ready)	58 (54,62)	2 (0, 4)	99 (98, 100)
Decullier, 2006^3^ [22]	Published as a scientific paper	6 years	47 (37,57)	2 (0, 6)	98 (95, 100)
Hahn, 2002 [26]	Published article or a written study report	5 years	*		
Chan, 2004a^3^ [18]	Journal article	4 years	31 (22,40)	1 (0, 3)	97 (91, 100)
Chan, 2004b^3^ [17]	Journal article	8 years	56 (50, 62)	2 (0, 5)	99 (97, 100)
Ghersi, 2006 [25]	NI	NI	NI		
Von Elm, 2008 [29]	Journal article	8 years	*		
Turer 2007 [28]	Peer reviewed journal articles	7 years	26 (20,33)^4^	*	*
De jong 2010 [21]	An original article containing study results, indexed in MEDLINE, EMBASE, PsychINFO, or the Cochrane Library	6 months to 9 years	NI		
Blumle 2008 [31]	Articles published in scientific journals that provide adequate information on at least the objectives of the study as well as on its methods and results	7 years	*		
Hall 2007 [32]	Peer reviewed journal	10–11 years	NI		

1 Denominator is the total number of studies with known publication status in the cohort i.e. published plus unpublished plus in preparation plus in press.

2 Denominator is the number submitted.

3 Denominator includes ongoing studies.

4 This is the number never presented and we have assumed not submitted.

* Question not asked. NI No information available.

The main findings of the empirical studies are shown in Table 5 and they are separated into study level and outcome level results. Nine of the included cohort studies [3], [4], [7], [19], [20], [23], [24], [30], [32] investigated results in relation to their statistical significance. One empirical study considered the importance of the results as rated by the investigator [22] and another empirical study considered confirmatory versus inconclusive results [7]. Five of the empirical studies [3], [4], [7], [23], [24] that examined the association between publication and statistical significance found that studies with statistically significant results were more likely to be published than those with non-significant results. Stern et al [4] reported that this finding was even stronger for their subgroup of clinical trials (Hazard Ratio (HR) 3.13 (95% confidence interval (CI) 1.76, 5.58), *p* = 0.0001) compared to all quantitative studies (HR 2.32 (95% CI 1.47, 3.66), *p* = 0.0003). One empirical study [19] found that studies with statistically significant results were more likely to be submitted for publication than those with non-significant results. Easterbrook et al [24] also found that study publication bias was greater with observational and laboratory-based experimental studies (Odds Ratio (OR) 3.79, 95% CI; 1.47, 9.76) than with RCTs (OR 0.84, 95% CI; 0.34, 2.09). Hall et al [32] found no difference in publication success in high impact journals i.e., >5 for trials reporting statistically significant or non-significant results (RR 0.929; 95% CI 0.759–1.137; *P = *0.537). However, two empirical studies [20], [30] found no statistically significant evidence for study publication bias (RR 4 (95% CI 0.6, 32) *p* = 0.1 and OR 0.53 (95% CI 0.25, 1.1) *p* = 0.1).

**Table 5:** Comparisons of primary outcome stated in protocol and in publication.

Study	Study level	Outcome level								
	Are studies with statistically significant or positive results, more likely to be published than those finding no difference between the study group?	Primary outcome stated in protocol is the same as in the publication	Primary outcome stated in protocol is downgraded to secondary in the publication	Primary outcome stated in the protocol is omitted from the publication	A non primary outcome in the protocol is changed to primary in the publication	A new primary outcome that was not stated in the protocol (as primary or secondary) is included in the publication	The definition of the primary outcome was different in the protocol compared to the publication	Completeness of reporting	Overall	Definition of disagreement/discrepancy
Easterbrook, 1991 [24]	OR 2.32, 95% CI; 1.25, 4.28.	*	*	*	*	*	*	*	*	*
Dickersin, 1992 [23]	OR 2.54, 95% CI; 1.63, 3.94	*	*	*	*	*	*	*	*	*
Dickersin, 1993 [3]	OR 12.30, 95% CI; 2.54, 60	*	*	*	*	*	*	*	*	*
Stern, 1997 [4]	HR 2.32, 95% CI; 1.47, 3.66, *p = *0.0003	*	*	*	*	*	*	*	*	*
Cooper, 1997 [19]	*p<*0.0001 (submission only)	*	*	*	*	*	*	*	*	*
Wormald, 1997 [30]	RR 4, 95% CI; 0.6, 32, *p* = 0.10	*	*	*	*	*	*	*	*	*
Ioannidis, 1998 [5]	*	*	*	*	*	*	*	*	*	*
Pich, 2003 [27]	*	*	*	*	*	*	*	*	*	*
Cronin, 2004 [20]	OR 0.53, 95% CI; 0.25, 1.1 *p* = 0.1	*	*	*	*	*	*	*	*	*
Decullier, 2005^2^ [7]	OR 4.59, 95% CI; 2.21, 9.54	*	*	*	*	*	*	*	*	*
Decullier, 2006 [22]	OR 1.58, 95% CI; 0.37, 6.71	*	*	*	*	*	*	*	*	*
Hahn, 2002 [26]	*	40% (6/15) stated which outcome variables were of primary interest and 4 of these (67% ) showed consistency in the reports	*	17% (1/6)	*	17% (1/6)	*	*	*	NA
Chan, 2004a^1^ [18]	*	67% (32/48)	23% (11/48)	13% (6/48)	9% (4/45)	18% (8/45)	36 trials reported a power calculation based on a particular outcome in their publications; 2 used an outcome that differed from the one used in the protocol and another introduced a power calculation that had not been mentioned in the protocol	Primary outcomes were incompletely reported in 7 (16%) of 45 trials that defined such outcomes in their publications	40% (19/48) of the trials contained major discrepancies in the specification of primary outcomes between the protocols and publications. None of the publications stated that an amendment had been made to the protocol.	The failure to report a pre-specified primary outcome; reporting of a prespecified primary outcome as secondary or as neither primary nor secondary in the publication; the introduction of new primary outcomes in the publication; and changing the outcome specified for the power calculation.
Chan, 2004b^1^ [17]	*	47% (36/76)	34% (26/76)	26% (20/76)	19% (12/63)	17% (11/63)	38 trials reported a power calculation, but 4 calculations were based on an outcome other than the one used in the protocol. In another 6 cases, there was a power calculation presented in a published article but not in the protocol	Primary outcomes were specified for 63 of the published trials, but for 17 (27%) of these trials at least one primary outcome was incompletely reported.	62% (51/82) of trials had major discrepancies between primary outcomes specified in protocols and those defined in the published articles (at least 1 primary outcome that was changed, introduced or omitted).	A prespecified primary outcome was reported as secondary or was not labelled as either; a prespecified primary outcome was omitted from the published articles; a new primary outcome was introduced in the published articles and the outcome used in the power calculation was not the same in the protocol and the published articles.
Ghersi, 2006 [25]	*	74%	*	31%	*	10%	60% definition the same, 31% unable to judge and 8% discrepant	The odds of fully reporting a comparison were greater if the result was statistically significant, (OR 2.2, 95% CI; 0.9–5.3, *p* = 0.08)	*	NA
Von Elm, 2008 [29]	*	*	*	26% (24/92)	*	11% (11/101)	*	*	*	NA
Turer 2007 [28]	*	*	*	*	*	*	*	*	*	*
De jong 2010 [21]	*	*	*	*	*	*	*	*	*	*
Blumle 2008 [31]	*	*	*	*	*	*	*	*	*	*
Hall 2007 [32]	*	*	*	*	*	*	*	*	*	*

NA Not Applicable.

* The study did not investigate this.

1 Gives results for trials with discrepancies for ≥1 primary outcome.

2 Results obtained from 248 of the completed studies.

Ioannidis et al [5] found that positive trials were submitted for publication more rapidly after completion than negative trials (median 1 vs 1.6 years, *p*<0.001) and were published more rapidly after submission (median 0.8 vs 1.1 years, *p*<0.04). Stern el al [4] and Decullier et al [7] also considered time to publication and found that those studies with positive results were published faster than those with negative results (median 4.8 v 8.0 years [4] and HR 2.48 (95% CI 1.36, 4.55) [7], respectively). However, for 53 trials where data were available, Hall et al [32] also found that there was no difference in the time to publication for trials reporting statistically significant results *vs* those reporting non-significant results (32±16 *vs* 36±24 months; mean ± SD; *P = *0.869).

Pich et al [27] looked at whether studies in their cohort were completed and published; 64% (92/143) of initiated trials were finished in accordance with the protocol and 31% (38/123) were published (or in-press) in peer reviewed journals. The main objective of the study by Blumle et al [31] was to consider how eligibility criteria stated in protocols was reported in subsequent reports, in doing so they noted that 52% of studies in their cohort were published, decreasing to 48% for RCTs only. Turer et al [28] looked at publication rates and found that 47% of studies in their cohort had been published. de Jong et al [21] aimed to identify prognostic indicators of the publication rate of clinical studies and found that 29% of studies had been published, although some had only been approved 6 months previously.

Seven empirical studies [3], [7], [19], [22]–[24], [30] described reasons why a study was not published as reported by the trialists. Reasons related to trial results included: unimportant/null results; results not interesting; results not statistically significant.

#### Outcome reporting bias

The total number of studies published in each cohort varied from 37% to 67% (Table 3). However, none of the empirical studies investigating ORB considered the proportions of published trials with positive, negative, or null overall results.

Table 4 shows that three of the empirical studies [17], [18], [29] defined ‘published' as a journal article; one empirical study [26] considered grey literature in their definition of ‘published’ although information on full publications and grey literature publications are separated (Figure 15). Although not considered in the definition of ‘published’, one empirical study [18] gave information on the grey literature or reports in preparation. Only two empirical studies [17], [18] present results for the percentage of studies not submitted (31% to 56%), the percentage of studies submitted but not accepted (1 to 2%) by the time of analysis of the cohort and the percentage of studies not published that were not submitted (97% to 99%).

All four empirical studies [17], [18], [25], [29] that examined the association between outcome reporting bias (outcome level bias) and statistical significance found that statistically significant outcomes were more likely to be completely reported than non-significant outcomes (range of odds ratios: 2.2 to 4.7 (Table 5)).

Five empirical studies [17], [18], [25], [26], [29] compared the protocol and the publication with respect to the primary outcome (Table 5). Only two empirical studies looked at the different types of discrepancies that can arise [17], [18] and concluded that 40–62% of trials had major discrepancies between the primary outcomes specified in protocols and those defined in the published articles. Four of the included empirical studies found that in 47–74% of studies the primary outcome stated in the protocol was the same as in the publication; between 13 and 31% of primary outcomes specified in the protocol were omitted in the publication and between 10 and 18% of reports introduced a primary outcome in the publication that was not specified in the protocol.

Chan et al also looked at efficacy and harm outcomes and in their Canadian empirical study [18] found that a median of 31% of efficacy outcomes and 59% of harm outcomes were incompletely reported and statistically significant efficacy outcomes had a higher odds than non significant efficacy outcomes of being fully reported (OR 2.7; 95% CI 1.5, 5). In their Danish empirical study [17] they found that 50% of efficacy and 65% of harm outcomes per trial were incompletely reported and statistically significant outcomes had a higher odds of being fully reported compared with non significant outcomes for both efficacy (OR 2.4, 95% CI; 1.4, 4) and harm (OR 4.7, 95% CI; 1.8, 12) data.

von Elm et al [29] considered efficacy and harm outcomes as well as primary outcomes overall and found that 32% (223/687) were reported in the publication but not specified in the protocol and 42% (227/546) were specified in the protocol but not reported, however this is preliminary data.

Two empirical studies [17], [18] describe the reasons why outcomes do not get reported but the study is published, these include lack of clinical importance and lack of statistical significance.

## Discussion

The four newly identified empirical studies only examined study publication bias. Outcome reporting bias was considered in one of the cohorts but results have only just been submitted for publication [31].

Very few of the 20 empirical studies examined both study publication bias and outcome reporting bias in the same cohort. Twelve of the included empirical studies demonstrate consistent evidence of an association between positive or statistically significant results and publication. They suggest that studies reporting positive/statistically significant results are more likely to be published and that statistically significant outcomes have higher odds of being fully reported.

In this review we focused on empirical studies that included RCTs since they provide the best evidence of the efficacy of medical interventions [60]. RCTs are prone to study publication bias, but it has been shown that other types of studies are more prone to study publication bias [24]. The main limitation of this review was that for eleven of the 20 included cohorts, information on RCTs could not be separated from information on other studies. Due to this barrier, and variability across empirical studies in the time lapse between when the protocol was approved and when the data were censored for analysis, we felt it was not appropriate to combine statistically the results from the different cohorts. Also, the fact that in six empirical studies [3], [4], [7], [21], [22], [24] follow-up of trials was less than 90% could mean that the problem of study publication bias is underestimated in these cohorts.

It is difficult to tell the current state of the literature with respect to study publication bias, as even the most recently published empirical evaluations included in the review, considered RCTs which began 10 years ago. Nevertheless, the empirical studies that were published within the last ten years show that the total amount of studies published was less than 50% on average.

None of the empirical studies explored the idea of all outcomes being non-significant versus those deemed most important being non-significant. In the reasons given, it was not stated which outcomes/how many outcomes were non-significant. Some empirical studies imply that all results were non-significant although this is due to the way the reason was written i.e. no significant results; but it is not explained whether this means for all outcomes, or primary and secondary, harm and efficacy etc. This implies a potential ambiguity of ‘no significant results’. It is not clear whether studies remain unpublished because all outcomes are non-significant and those that are published are so because significant results are selectively reported. This is where study publication bias and outcome reporting bias overlap.

Dubben et al [61] looked at whether study publication bias exists in studies which investigate the problem of study publication bias. Although they found no evidence of study publication bias, it is interesting to note that two of the included cohorts in this review have not been published [25], [30]. The study conducted by Wormald et al [30] concluded that ‘there was limited evidence of study publication bias’ whereas the authors of the other study [25] have not submitted their study for publication. There may be other unpublished studies of study publication bias or outcome reporting bias that were not located by the search, however contact with experts in the field reduces the likelihood of these issues introducing bias.

Submission is an important aspect of investigating study publication bias as it will provide information on whether reports are not being published because they are not submitted or they are submitted but not accepted. Obviously those studies that are not submitted are not published and it was found by Dickersin et al [62] that non-publication was primarily a result of failure to write up and submit the trial results rather than rejection of submitted manuscripts. This is confirmed for the cohorts identified here with the percentage of studies not published due to not being submitted ranging from 63% to 100%. Olson et al [63] also found that there was no evidence that study publication bias occurred once manuscripts had been submitted to a medical journal. However, this study looks at a high impact general journal, which is unlikely to be representative for specialist journals that publish the majority of clinical trials.

Eleven studies assessed the impact of funding on publication; this was done in several ways. Three studies found that external funding lead to a higher rate of publication [4], [22], [23]. von Elm et al [29] found that the probability of publication decreased if the study was commercially funded and increased with non commercial funding. Easterbrook et al [24] found that compared with unfunded studies, government funded studies were more likely to yield statistically significant results but government sponsorship was not found to have a statistically significant effect on the likelihood of publication and company sponsored trials were less likely to be published or presented. Dickersin et al [3] found no difference in the funding mechanism grant versus contract and Ioannidis et al [5] found no difference in whether data were managed by the pharmaceutical industry or other federally sponsored organisations. Chan 2004b et al [17] found that 61% of the 51 trials with major discrepancies were funded solely by industry sources compared with 49% of the 51 trials without discrepancies. Ghersi [25] did examine the effect of funding in terms of reporting and discrepancies of outcomes but no information about the results is currently available. Hahn et al [26] compared the funder stated in protocol to publication. Hall et al [32] found that studies sponsored by the pharmaceutical industry were less likely to be published than those sponsored by federal granting agencies (RR 0.50; 95% CI 0.39–0.65; *P = *0.0045) but were more likely to be published than studies funded by the local health authority (RR 1.94; 95% CI 1.09–3.44; *P = *0.011). These studies indicate that funding is an important factor to consider when investigating publication bias and outcome reporting bias, however more work needs to be done to examine common questions before conclusions regarding the relationship between funding and outcome reporting bias can be drawn.

Our review has examined inception cohorts only, however, other authors have investigated aspects of study publication bias and outcome reporting bias using different study designs, with similar conclusions. Since the original version of this review was published [13], a Cochrane methodology review is now available on publication bias [64]. This review included five studies, only one of which was not included in our review as it was not an inception cohort [40]. Hopewell et al concluded that trials with positive findings are published more often and more quickly than trials with negative findings [64]. The Cochrane review by Scherer et al [6] investigating the full publication of results initially presented in abstracts found that only 63% of results from abstracts describing randomized or controlled clinical trials are published in full and ’positive’ results were more frequently published than non ’positive’ results. Several studies investigated a cohort of trials submitted to drug licensing authorities [38], [40], [41], [65] and all found that many of these trials remain unpublished, with one study demonstrating that trials with positive outcomes resulted more often in submission of a final report to the regulatory authority [40]. Olson et al [63] conducted a prospective cohort study of manuscripts submitted to JAMA and assessed whether the submitted manuscripts were more likely to be published if they reported positive results. They did not find a statistically significant difference in publication rates between those with positive and negative results. None of the inception cohorts addressed the question as to whether the significance determined whether a submitted paper was accepted or not, with the exception of one inception cohort [5] that found that “positive” trials were published significantly more rapidly after submission than “negative” trials. Finally, a comparison of the published version of RCTs in a specialist clinical journal with the original trial protocol found that important changes between protocol and published paper are common; the published primary outcome was exactly the same as in the protocol in six out of 26 trials (23%) [50] This was also highlighted in a recent Cochrane methodological review [16], which included 12 studies comparing protocols to published reports and four studies comparing trial registry entries to published reports.

We recommend that researchers use the flow diagram presented in this work as the standard for reporting of future similar studies that look at study publication bias and ORB as it clearly shows what happens to all trials in the cohort.

Reviewers should scrutinise trials with missing outcome data and ensure that an attempt to contact trialists is always made if the study does not report results. An outcome matrix generator has now been developed as a tool to help identify missing outcome data at the study level within a review (http://ctrc.liv.ac.uk/orbit/). Also, the lack of reporting of specified outcome(s) should not be an automatic reason for exclusion of studies. Statisticians should be involved for the data extraction of more complex outcomes, for example, time to event. Methods that have been developed to assess the robustness of the conclusions of systematic reviews to ORB [66]–[68] should be used. Meta-analyses of outcomes where several relevant trials have missing data should be seen with extra caution. In all, the credibility of clinical research findings may decrease when there is wide flexibility in the use of various outcomes and analysis in a specific field and this is coupled with selective reporting biases.

The setting up of clinical trials registers and the advanced publication of detailed protocols with an explicit description of outcomes and analysis plans should help combat these problems, although it should be noted that other work has shown that there can be discrepancies between protocols/trial registries and published reports [16]. Trialists should be encouraged to describe legitimate changes to outcomes stated in the protocol.

For empirical evaluations of selective reporting biases, the definition of significance is important as is whether the direction of the results is taken into account, i.e. whether the results are significant for or against the experimental intervention. However, only one study took this into account [5]. The selective publication preference forces may change over time. For example, it is often seen that initially studies favouring treatment are more likely to be published and those favouring control suppressed. However, as time passes, contradicting trials that favour control may become attractive for publication, as they are ‘different.’ The majority of cohorts included in this review do not consider this possibility.

Another recommendation is to conduct empirical evaluations looking at both ORB and study publication bias in RCTs to investigate the relative importance of both i.e. which type of bias is the greater problem. The effects of factors such as funding, i.e. the influence of pharmaceutical industry trials versus non pharmaceutical trials, should also be factored in these empirical evaluations.

## Supporting Information

Table S1 **PRISMA 2009 Checklist.**
(DOC)

Appendix S1 **Search Strategy.**
(DOC)

Text S1 **Explanation of flow diagram.**
(DOC)
